# Clarifying the Ghrelin System’s Ability to Regulate Feeding Behaviours Despite Enigmatic Spatial Separation of the GHSR and Its Endogenous Ligand

**DOI:** 10.3390/ijms18040859

**Published:** 2017-04-19

**Authors:** Alexander Edwards, Alfonso Abizaid

**Affiliations:** Department of Neuroscience, Carleton University, 1125 Colonel By Drive, Ottawa, ON K1S 5B6, Canada; alexander.edwards@carleton.ca

**Keywords:** feeding, ghrelin, GHSR, blood brain barrier, vagal afferents, circumventricular organs, central ghrelin synthesis, GHSR heterodimerization, GHSR constitutive activity

## Abstract

Ghrelin is a hormone predominantly produced in and secreted from the stomach. Ghrelin is involved in many physiological processes including feeding, the stress response, and in modulating learning, memory and motivational processes. Ghrelin does this by binding to its receptor, the growth hormone secretagogue receptor (GHSR), a receptor found in relatively high concentrations in hypothalamic and mesolimbic brain regions. While the feeding and metabolic effects of ghrelin can be explained by the effects of this hormone on regions of the brain that have a more permeable blood brain barrier (BBB), ghrelin produced within the periphery demonstrates a limited ability to reach extrahypothalamic regions where GHSRs are expressed. Therefore, one of the most pressing unanswered questions plaguing ghrelin research is how GHSRs, distributed in brain regions protected by the BBB, are activated despite ghrelin’s predominant peripheral production and poor ability to transverse the BBB. This manuscript will describe how peripheral ghrelin activates central GHSRs to encourage feeding, and how central ghrelin synthesis and ghrelin independent activation of GHSRs may also contribute to the modulation of feeding behaviours.

## 1. Introduction

### 1.1. Preface

**Figure 1:**
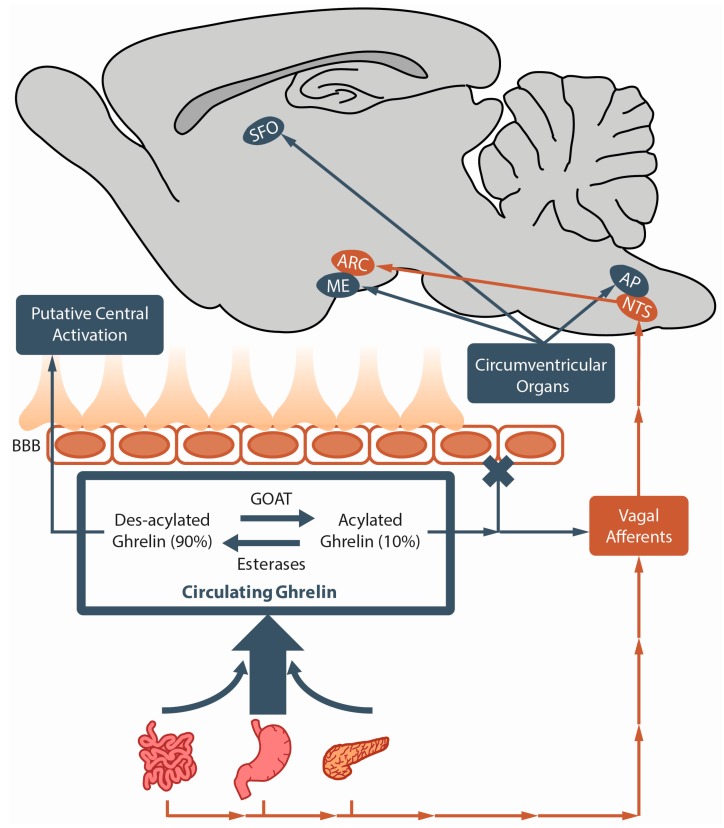
Illustration describing how peripherally produced ghrelin likely gains access to the brain. Ghrelin is primarily produced and secreted by the stomach although the small intestine and pancreas likewise produce a small quantity. Ghrelin *O*-acyltransferase (GOAT) converts des-acylated into its active acylated form capable of activating growth hormone secretagogue receptor (GHSRs) while esterases cleave acylated ghrelin’s *O*-octanoyl moiety returning it to its predominant inactive des-acylated ghrelin form (90% of total circulating ghrelin). Although des-acylated ghrelin is capable of crossing the blood brain barrier from the blood into the brain, acylated ghrelin demonstrates a very limited ability to do so (depicted by blue X). Accordingly, acylated ghrelin either stimulates GHSR on vagal afferents or bypasses the blood brain barrier (BBB) and activates GHSRs in or around circumventricular organs to convey its orexigenic effects. AP, area postrema; ARC, arcuate nucleus; ME, median eminence; NTS, nucleus tractus solitaries; SFO, subfornical organ.

Ghrelin is a 28 amino acid peptide hormone that influences many physiological processes (adiposity, anxiety, feeding behaviours, memory, etc.) by binding and activating its endogenous seven transmembrane G protein couple receptor (GPCR): The type 1a growth hormone secretagogue receptor (GHSR) [1,2,3,4,5,6,7,8,9,10]. As one would expect given the multifaceted nature of ghrelin, GHSRs are widely distributed within the brain (e.g., hypothalamus (HYP), ventral tegmental area (VTA), hippocampus (HIP)) as well as in peripheral organs (e.g., adipose tissue, adrenals, stomach) [11,12,13,14,15,16,17]. However, the ghrelin peptide, which is predominantly produced and secreted into the bloodstream by endocrine gastric cells of the stomach, demonstrates a very limited ability to cross the blood brain barrier (BBB) from the circulation into the brain (refer to Figure 1) [1,3,18,19,20,21]. Although some insist that ghrelin is likewise produced in the brain, the validity of its central synthesis is a topic of contention [22,23,24,25]. Given its predominant peripheral production and poor ability to cross the BBB, one of the most pressing unanswered questions plaguing ghrelin research is how GHSRs, which are widely distributed in brain regions protected by the BBB, are activated to modulate ghrelin attributed processes and behaviours. This apparent paradox has not been adequately addressed, an alarming fact considering the vast amount of resources that have been spent characterizing central GHSR dependent behaviours. Moreover, the relevance and applicability of many ghrelin system studies heavily rely on reconciling this knowledge gap. Accordingly, this paper discusses the most plausible means by which central GHSRs are activated to modulate behaviour and highlights crucial studies that are needed to clarify these mechanisms. As the ghrelin system’s ability to regulate feeding behaviours was recognized shortly after ghrelin’s discovery and is perhaps the most renown; how the ghrelin system promotes feeding behaviours despite the enigmatic spatial separation of GHSR and its endogenous ligand will be the focus of this review.

### 1.2. Ghrelin: The Feeding Peptide

Ghrelin is distinct from most peripherally produced feeding related signals as it is one of the only ones that stimulates food intake [6,9,10]. Dubbed as “the feeding peptide”, its circulating levels fluctuate with feeding status (high in the fasted state and low post-prandially) and oscillate in time with scheduled meals [12,18,26,27,28,29,30,31,32,33]. Consistent with this, exogenous ghrelin induces feeding when administered peripherally (i.e., intraperitoneal or intravenous) or centrally (i.e., intracerebroventricular) [1,6,9,10,27,34]. Interestingly, ghrelin is even capable of eliciting feeding during times of positive energy balance [6]. Conversely, GHSR antagonists block feeding traditionally observed following ghrelin injections in ad libitum fed mice [1,6,35]. Likewise, GHSR antagonists are capable of reducing food intake in food deprived rodents when endogenous levels of ghrelin are high [36,37]. This in conjunction with the fact that ghrelin does not enhance feeding in GHSR null mice strongly suggests that ghrelin enhances food intake via GHSR dependent processes [8,38].

The protein product of the ghrelin gene requires post translational modifications to generate the active form of the ghrelin peptide (acyl-ghrelin) [39]. The pre-pro ghrelin peptide is cleaved into two biologically active peptides, obestatin and des-acyl ghrelin, both of which appear to have anorectic effects [39,40,41,42,43]. It has been suggested that obestatin may specifically inhibit ghrelin induced food intake as many fail to see anorectic effects following administration of obestatin alone [44,45,46,47,48]. Des-acyl ghrelin is further modified (i.e., acylated) by ghrelin *O*-acyltransferase (GOAT) [6,8,20,49,50,51]. This modification, generally an octanoylation, occurs on serine-3 of the peptide and is required for the orexigenic activity of ghrelin [50,51]. The addition of this side chain induces a conformational alteration in the peptide that allows ghrelin to access the active binding site of the GHSR [49,50,51]. Interestingly, the *O*-octanoyl moiety also makes acylated ghrelin much more hydrophobic [52]. This hydrophobicity facilitates interactions between acylated ghrelin and the cellular membrane to encourage receptor binding [52]. GOAT mRNA is ubiquitously expressed within many peripheral organs (e.g., adrenal cortex, spleen, lungs, etc.) but is typically highest in organs that also strongly express ghrelin mRNA (e.g., stomach and intestines) [50,53]. Like ghrelin, GOAT expression also fluctuates in response to feeding status and diet [30,50,54,55,56,57]. Although initial studies demonstrated negligible expression of GOAT in the brain, GOAT appears to be modestly expressed in the HYP, a brain region that is sensitive to ghrelin and also credited with the putative ability to produce ghrelin [22,24,50,54,56].

Astonishingly, it is estimated that a mere 10% of circulating ghrelin is acylated and thus capable of binding GHSRs to induce feeding [58]. While recent data suggest that the proportion of acyl ghrelin may be higher, des-acyl ghrelin remains the principal form of ghrelin in blood [59]. Des-acyl ghrelin does not seem to increase GHSR signalling at physiological concentrations [11,20,60,61] but see [62]. It has been proposed that supraphysiological concentrations of des-acylated ghrelin are needed to bind GHSRs as des-acylated ghrelin lacks the *O*-octanoyl hydrophobic side chain that acylated ghrelin uses to anchor itself in membranes [52,62]. Accordingly, des-acyl ghrelin is much less likely to encounter and stay associated with cellular membranes and thus less likely to interact with GHSRs [52]. As mentioned above des-acyl ghrelin has been reported to influence feeding behaviours; however, the data is inconsistent and is hypothesized to be mediated through a GHSR independent mechanism [63].

### 1.3. Ghrelin’s Hypothalamic Modulation of Feeding

The HYP, which can be subdivided into many distinct nuclei such as the paraventricular nucleus (PVN), lateral hypothalamic area (LHA), dorsomedial nucleus (DMH), ventromedial nucleus (VMN), and the arcuate nucleus (ARC), is responsible for regulating homeostatic feeding and energy balance [13,14,16,22,24,64,65,66]. All of these major hypothalamic nuclei express GHSRs and demonstrate the capacity to initiate food intake in response to local ghrelin microinfusion [10,17,22,67,68,69]. The ARC, which sits adjacent to the third ventricle and the median eminence (i.e., an important circumventricular organ), is especially important as it has receptors for and is sensitive to most circulating hormones that influence energy balance, including ghrelin [10,17,21,22,67,68,69,70,71,72,73,74] but see [75].

**Figure 2:**
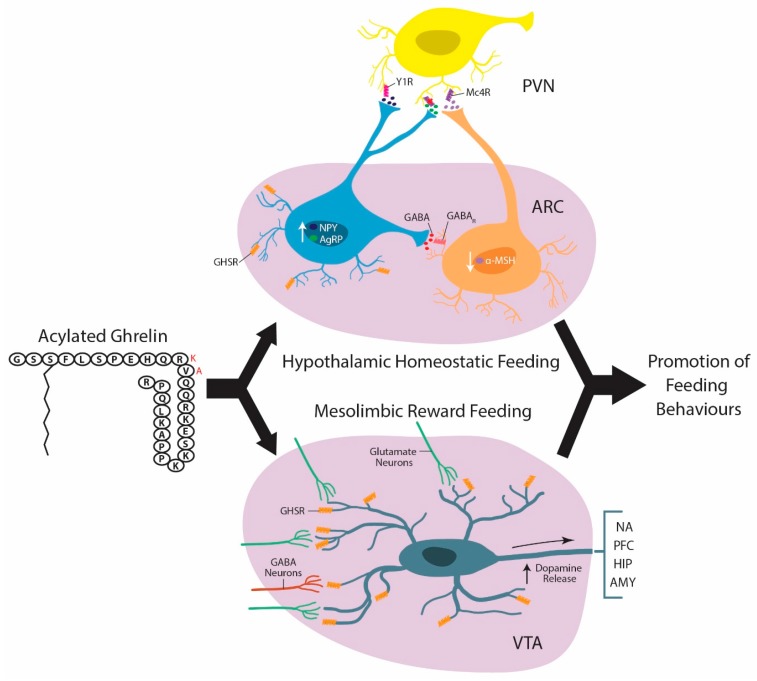
Over simplified illustration depicting the two main brain regions where acylated ghrelin (human ghrelin is black amino acid sequence and red letter substitution is rat) is proposed to target to initate neurocircuits that promote feeding behaviours: the arcuate nucleus (ARC) of the hypothalamus (HYP) and the ventral tegmental area (VTA). Within the ARC, ghrelin stimulates neuropeptide Y/agouti-related peptide (NPY/AGRP) neurons by binding growth hormone secretagogue receptors (GHSRs) on their surface. Once activated theses neurons produce and release γ-aminobutyric acid (GABA) which inhibits anorectic proopiomelanocortin (POMC) neurons,decreasing the release of the anorectic peptide α-melanocyte-stimulating hormone (α-MSH). This effectively reduces the quantity of α-MSH capable of binding to satiety promoting melanocortin 4 receptors (Mc4Rs). Concurrently, activated NPY/AGRP neurons increase their production and secretion of orexigenic peptides NPY and AGRP. NPY binds to neuropeptide Y receptor type 1 (Y1R) and AGRP antagonizes the binding of α-MSH at Mc4Rs. Together the reduction in anorectic peptide and enhancement of orexigenic ones work to reduce the activity of second order anorexigenic neurons in the paraventricular nucleus (PVN) to promote homeostatic feeding behaviours. Similarly, ghrelin also stimulates VTA dopamine (DA) neurons increasing the frequency and probability of DA release from their projections in the nucleus accumbens (NA), prefrontal cortex (PFC), hippocampus (HIP), and amygdala (AMY) to encourage mesolimbic reward feeding. Ghrelin activates these VTA dopamine neurons both directly by binding to GHSR receptors located on their surface and indirectly by increasing the ratio of excitatory to inhibitory synapses contacting them.

Accordingly, the ARC demonstrates increased c-Fos staining following central or peripheral administration of ghrelin [76,77,78].Consistent with this, lesioning the ARC prevents the typical increase in food intake that follows either central or peripheral ghrelin administration [79,80]. Not surprisingly, GHSR are expressed in the two major opposing cell types that regulate homeostatic feeding, neuropeptide Y/agouti-related peptide/γ-aminobutyric acid (NPY/AGRP/GABA) and to a lesser extentin proopiomelanocortin/cocaine and amphetamine regulated transcript neurons (POMC/CART) [65]. Accordingly, ghrelin upregulates the expression of orexigenic peptide transcripts, NPY and AGRP, but does not upregulate the anorectic peptide POMC within the ARC [81]. More importantly, although ghrelin binding studies show that ghrelin targets both of these cell types, the direct effect of ghrelin on POMC/CART neurons has not be well characterized [21,22]. NPY/AGRP/GABA neurons enhance appetite when activated whereas POMC/CART neurons promote satiety when stimulated [22,82,83,84,85]. Ghrelin enhances the activity of NPY/AGRP/GABA neurons by both binding to GHSRs on their surface and by increasing the ratio of excitatory to inhibitory afferents contacting them [22,86,87,88]. Moreover, the proportion of NPY/AGRP/GABA neurons targeted by ghrein is higher when animals are fasted as one would expect when food procuring behaviours are needed [21]. Conversely, ghrelin inhibits POMC/CART neurons by decreasing the proportion of excitatory to inhibitory afferents innervating them and by directly increasing GABA release from NPY/AGRP/GABA onto them [22,86,87,88]. Once activated NPY/AGRP/GABA neurons stimulate feeding by increasing the production and release of NPY, AGRP, and GABA, which together act to turn down satiety promoting pathways (Figure 2) [22,89,90,91,92]. As mentioned, GABA released from NPY/AGRP/GABA neurons directly inhibits neighbouring POMC/CART neurons [22]. Second, AGRP antagonizes the binding of POMC/CART neuron released α-melanocyte-stimulating hormone (α-MSH) and β-MSH at melanocortin receptors (i.e., MC_4_) located in the PVN, LHA, and DMH, to dampen anorectic outputs from these regions [82,89,90,93,94,95,96]. Lastly, the enhanced production and release of NPY from NPY/AGRP/GABA projections innervating many other hypothalamic nuclei (i.e., PVN, VMH, and LHA) likewise encourages appetite [64,89,91,96,97,98]. On the other hand, shortly following meal consumption ghrelin levels drop whereas nutrient and hormonal satiety signals rise [86]. Consequently, NPY/AGRP/GABA neuron activity drops off resulting in reduced inhibitory (i.e., reduced GABA tone) control over POMC/CART neurons [22]. In addition, POMC/CART neuron secreted anorectic peptides, α-MSH and β-MSH, are less antagonized by AGRP at melanocortin receptors in the aforementioned regions of the HYP [64]. Likewise, NPY production and secretion from NPY/AGRP/GABA neurons is attenuated [64]. Ultimately, the heightened production of α-MSH, β-MSH, and CART from POMC neurons and reduced NPY/AGRP/GABAergic activity leads to dampening of orexigenic pathways and the manifestation of satiety [64].

### 1.4. Ghrelin’s Hedonic Regulation of Feeding

In addition to its role in promoting homeostatic feeding, within the HYP, ghrelin also influences motivational and hedonic aspects of feeding behaviours [1,99,100,101]. Accordingly, administration of ghrelin increases while disruption of the ghrelin system using antagonists or GHSR knockout (KO) animals decreases the consumption of palatable foods [36,101,102,103,104,105,106]. Furthermore, ghrelin injections facilitate the development of conditioned place preferences (CPP) and enhance bar press break points for rewarding foods [37,107]. Conversely, GHSR antagonist treated and GHSR KO animals are unable to develop CPP and demonstrate decreased break points for palatable foods [37,107].

The mesolimbic dopaminergic system has long been implicated in modulating behaviours that are engaged whilst trying to acquire both natural (e.g., food) and drug rewards (e.g., cocaine) [1,108,109]. The activity of this system is driven primarily by a large population of dopamine neurons in the mid brain (i.e., VTA), and their projections to the nucleus accumbens (NA), amygdala (AMY), HIP, and prefrontal cortex (PFC) [108,109]. Lesion studies that attenuate dopamine release within this pathway highlight its importance in controlling feeding behaviours [110,111]. Accordingly, lesioned animals siginficantly decrease thier food intake and body weight [110,111]. Consistent with this, the incentive properties of palatable foods induce increases in dopamine release within projectional target regions such as the NA [112].

The VTA is one of the main brain regions where ghrelin acts to encourage hedonic and motivational food related behaviours (Figure 2) [1,101,113]. In support of this, appreciable levels of GHSR transcript are reported throughout the mesolimibic dompainergic system, especially within the VTA [1,13,17]. VTA lesioned animals are less motivated to search and bar press for food following central ghrelin infusions compared to controls [103,114,115]. Furthermore, intra-VTA infusions of ghrelin stimulate feeding and enhance the motivation to work for palatable foods, whereas intra-VTA ghrelin antagonists block ghrelin induced feeding and reduce motivation to obtain palatable food rewards [1,101,113,116]. Interestingly, administration of a ghrelin antagonist into the VTA is not only sufficent to reduce refeeding following a fast but also the motivation to obtain food rewards [1,101,113]. Ventricular infusions of ghrelin enhance phasic dopamine and NA neural activity in response to food predictive cues [117]. Moreover, bath application of ghrelin to VTA brain slices enhances VTA dopamine neuron activity [1] and intra-VTA microinfusions of ghrelin increases dopamine turnover in the NA [1,118,119]. Interestingly, peripheral ghrelin administration is sufficient to increase dopamine release within the nucleus accumbens following consumption of standard chow suggesting that peripheral ghrelin is likewise capabale of directly or indirectly activating VTA dopamine neurons [120]. The ability of ghrelin to enhance dopamine release within the mesolimbic dopamine system has been attributed to concomitant activation of GHSRs on VTA dopamine neurons and the enhancement of the ratio of excitatory versus inhibitory afferents contacting them (Figure 2) [1]. Importantly, the excitation of VTA dopamine neurons, enhanced dopamine turnover in the NA, and heightened feeding responses that occur in response to ghrelin administation are not observed in GHSR KO mice [1]. Lastly, when compared to GHSR KO animals, mice that solely express functional GHSRs on dopamine neurons, have much better CPP scores when responding for palatable rewards and greater acute food intake in response to ghrelin [121]. Collectively, these data exemplify the role that the ghrelin system plays in regulating motivated feeding behaviours, highlight the VTA as an integral brain region whereby ghrelin influences these behaviours, and demonstrate the importance of GHSRs in meditating these processes.

## 2. The Ghrelin and Growth Hormone Secretagogue Receptor Paradox

It is evident that ghrelin has the capacity to drive feeding related behaviours when injected directly into the brain (i.e., most nuclei of the HYP, HIP, and VTA). Furthermore, neurons within these regions express ghrelin receptors (transcript and protein) and are responsive to acyl ghrelin [1,4,11,13,14,17,22]. Paradoxically, ghrelin is widely accepted to be predominately produced in the periphery and to have a very limited capacity to cross the BBB [19,21,122]. It is clear that there is a disjunction between where central GHSRs are known to reside and ghrelin’s accessibility to these regions. The rest of this manuscript will seek to explain the most plausible means by which the ghrelin system encourages feeding through the activation of central GHSRs. As part of this, we discuss the putative role of peripherally secreted ghrelin, centrally synthesized ghrelin, and ghrelin independent GHSR activity in encouraging feeding behaviours.

### 2.1. Peripheral Ghrelin Activates Central Targets

**Figure 3:**
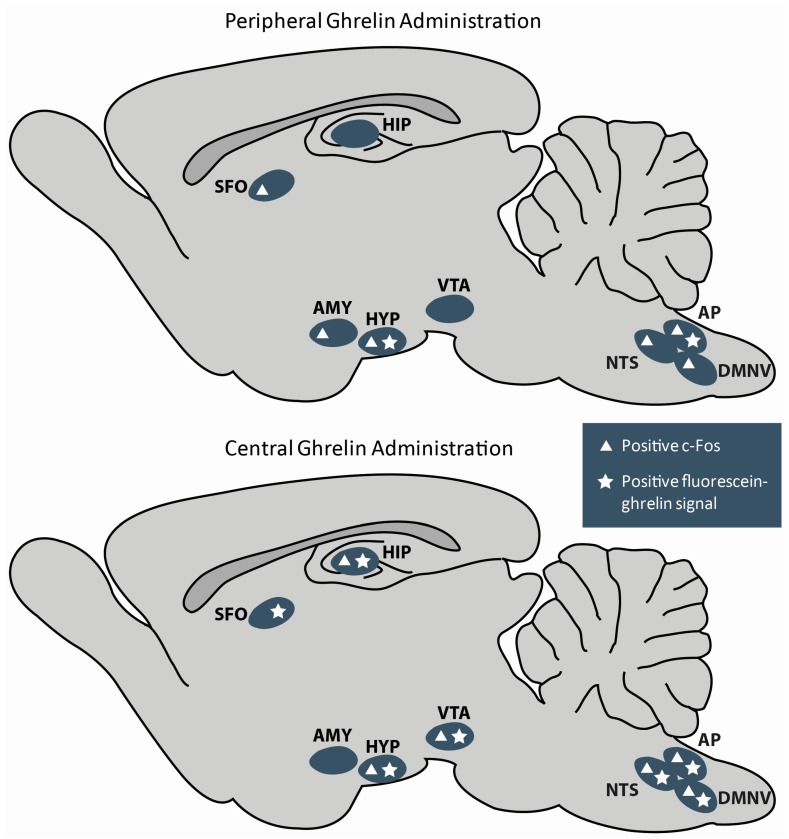
Illustration of the feeding related brain regions that demonstrate positive c-Fos and/or fluorescein-ghrelin signals following peripheral and central ghrelin administration. Fluorescein ghrelin is a probe that has been used to determine what brain regions normal acylated ghrelin accesses. It is a fluorescently tagged (fluorescein isothiocyanate) truncated analog of the ghrelin peptide (i.e., 18 amino acids) with equivalent receptor stability, agonist activity, and binding affinity as acylated ghrelin [133]. AMY, amygdala; AP, area postrema; DMNV, dorsal motor nucleus of the vagus nerve; HIP, hippocampus; HYP, hypothalamus; NTS, nucleus tractus solitaries; SFO, subfornical organ; VTA, ventral tegmental area.

Shortly after it was learned that the stomach supplies approximately 80% of total circulating ghrelin but that centrally administered ghrelin also increases feeding, efforts were made to determine if peripheral ghrelin was capable of activating central targets [6,18,20,28,123]. Accordingly, studies looking at c-Fos expression (a reliable indicator of neuronal activation) following peripheral ghrelin administration ensued (Figure 3) [124]. Early c-Fos experiments reported significant c-Fos expression within the ARC, PVN, and DMH, but negligible expression within other GHSR positive feeding related brain regions such as the hindbrain and remaining hypothalamic nuclei [13,17,78,123,125,126,127]. More recent studies, in addition to replicating data collected from early c-Fos studies, demonstrate considerable c-Fos expression in sensory circumventricular regions such as the subfornical organ (SFO) and area postrema (AP) as well as other brain regions that express GHSRs (i.e., amygdala, lateral parabrachial nucleus, nucleus tractus solitarius (NTS), and the dorsal motor nucleus of the vagus nerve (DMNV)) [13,17,122,128,129,130] but see [131]. The aforementioned c-Fos studies suggest that peripheral ghrelin can activate many BBB protected brain regions in addition to more accessible brain regions (i.e., ARC, AP, and SFO); however, they fail to describe whether ghrelin directly targets these BBB privileged regions. It is important to note that c-Fos expression can be upregulated in downstream activated cells of circuits influenced but not directly targeted by ghrelin. c-Fos experiments where ghrelin is peripherally injected into GHSR KO mice that have GHSRs selectively rescued in specific BBB-protected brain regions of interest should allow for the parsing out of direct versus indirect effects of peripheral ghrelin within these regions. Nonetheless, the fact that peripheral ghrelin is unable to stimulate feeding in transgenic mice that lack GHSRs in neurons substantiates the notion that peripheral ghrelin targets central GHSRs to induce feeding [132].

### 2.2. The Blood Brain Barrier

Although blood-cerebral spinal fluid, blood-labyrinth, blood-retina, blood-nerve and blood-brain barriers work together to regulate the transport of molecules into and out of the central nervous system, the latter is by far the largest and most commonly discussed blood-central nervous system interface [134]. Unlike the fenestrated capillaries ubiquitously found in the periphery, a vast majority of central nervous system capillaries are protected by the BBB. The BBB, which lines brain microvessels, is composed of endothelial cells connected by tight junctions [135,136]. The BBB works in close concert with neighbouring cells, mainly pericytes, smooth muscle cells, and astrocytes, to maintain a homeostatic neuroparenchymal microenvironment within the central nervous system [134,137]. In general, steroidal hormones (e.g., testosterone, estrogen) are able to cross the BBB by passive transmembrane diffusion due to their small size and lipophilic nature; whereas, larger peptide hormones (e.g., insulin, ghrelin) often require saturable specialized transporters to cross the BBB [138]. Accordingly, hormones that can enter the brain using non-saturable processes have similar blood and brain concentrations [139]. In contrast, the rate in which peptide hormones are transported by saturable mechanisms depends on the location and quantity of its corresponding transporter as well as its affinity for its respective ligand, frequently resulting in different brain and blood concentrations [138,140].

### 2.3. Permeability of the Blood Brain Barrier to Ghrelin

The capacity of ghrelin to transverse the BBB was comprehensively described in a seminal paper by Banks and colleagues [19], in which they tested the efficiency of three radioactively labelled ghrelin peptides (i.e., human, mouse, and mouse des-acyl ghrelin) to cross the mouse BBB in both the brain-to-blood and blood-to-brain direction. Reportedly, mouse acylated ghrelin is transported in a saturable fashion only in the brain to blood direction but not in the blood to brain direction; whereas, des-acyl ghrelin travels into the brain from the blood easily via nonsaturable diffusion yet does not travel from the brain into the blood easily [19]. Interestingly, human ghrelin, which differs in only 2 amino acids from that of mouse ghrelin, can cross the mouse BBB in both directions via saturable transport [19]. Consistent with this, human ghrelin also demonstrates both saturable binding and endocytosis in in vitro studies using rat cerebral microvessel endothelial cells (i.e., RBE4 cells) [141]. Interestingly, lipid bilayer studies demonstrate that acylated ghrelin is incapable of crossing lipid bilayers unaided suggesting that if peripheral ghrelin does access central brain regions it is either via circumventricular organs or through a currently unidentified specialized saturable transporter [52]. Modest and saturable radioactive signals can be detected in several regions of the mouse brain (i.e., olfactory bulbs, occipital cortex, HYP, and HIP) following peripheral administration of radioactively labelled human ghrelin [3,142]. Although using tagged human ghrelin to study the capacity of ghrelin to cross the BBB and its central targets in mice is valuable, the data should be interpreted with caution given that rodent ghrelin seems to have a very limited capacity to do so [19]. Moreover, although some have investigated ghrelin’s BBB permeability at physiologically relevant times when the BBB is considered less impenetrable and ghrelin levels are naturally high (i.e., fasted or food restricted animals) [21,142] most studies have not taken into account the nutritional status of their experimental rodents [3,19,122]. Accordingly, recent data, utilizing fluorescently labelled truncated forms of ghrelin, validated to have similar characteristics as normal acylated ghrelin (e.g., stability, receptor binding, agonist activity), suggest that peripheral ghrelin is only capable of accessing the ventromedial portion of the mouse HYP (i.e., ARC and median eminence) and that its capacity to do so may rely on the energy state of tested rodents (a more detail discussion of these studies is found elsewhere in this manuscript) [21,122,133].

One cannot ignore the possibility that mouse des-acyl ghrelin, which is more stable and capable of crossing the BBB from the blood into the brain of mice, is produced peripherally but activated centrally, especially given that the transcript for GOAT is found within the brain (i.e., HYP) and its central knockdown has overt behavioural effects [54,56]. In support of this, although early studies suggested endoplasmic reticulum residency of GOAT, GOAT protein has also been discovered in the circulation of rodents [51,143]. The fact that the enzyme GOAT is not exclusively found intracellularly hints at a putative extracellular mechanism of ghrelin acylation. Moreover, food restriction and fasting both cause a consequential increase in hypothalamic GOAT mRNA expression suggesting that this enzyme is upregulated and well situated to activate ghrelin during times of energy insufficiency (i.e., when endogenous ghrelin levels are high) [18,26,27,30,56]. To our knowledge no one has extensively characterized GOAT protein within the brain likely due to distrust of currently available polyclonal antibodies targeting GOAT. Discovery and isolation of a reliable GOAT antibody and subsequent analysis of GOAT protein expression within feeding nuclei and/or within the cerebrospinal fluid (CSF) needs to be completed before the notion of central activation of peripherally produced ghrelin is revisited.

It is unknown whether endogenous acylated ghrelin is capable of crossing the BBB in humans largely due to the invasiveness and limits of current techniques (e.g., brain microdialysis, CSF sampling) capable of probing this question. Likewise, although post-mortem analysis of animal tissue has provided some valuable information pertaining to the permeability of the brain to peripheral ghrelin, these studies are impractical and unethical to conduct in humans [3,19,21,122]. As a result, brain imaging studies (fMRI) have been widely adopted to investigate the effect of peripheral ghrelin on the human brain [144,145,146]. Although these studies demonstrate that peripheral ghrelin selectively activates particular feeding related brain regions, it remains unknown whether peripheral ghrelin directly or indirectly targets these regions [144,145,146]. Positron emission tomography (PET), an imaging technique that uses the decay of radioactive tracers attached to small biologically active molecules to explore the functionality of organs, may be useful in determining whether peripheral ghrelin directly targets central GHSRs. Fortunately, biologically active radioactive GHSR agonist tracers exist and may prove useful in this regard [147]. Accordingly, GHSR competition assays that compare PET signals following administration of these radioactively labelled GHSR agonists on their own to those obtained proceeding co-administration of them with human ghrelin, should help clarify whether and the extent to which peripheral ghrelin directly targets central GHSRs.

## 3. Peripheral Ghrelin Stimulates Appetite

### 3.1. Vagal Afferent Activation by Ghrelin

The vagus nerve (i.e., tenth cranial nerve) is integral for brain-gut communication. It mediates the transmission of neural signals from the brain to the gastrointestinal system and likewise conducts hormonal information from the gastroenteric system to the brain (Figure 1) [148,149,150]. Although the vagus nerve is comprised of both afferent and efferent nerve fibers; the vast majority (i.e., approximately 90%) of neurons are afferent connections to the NTS [151]. Accordingly, the NTS is the main brain region where feeding related afferent signals from the gastroenteric system are integrated with descending hypothalamic information prior to the transfer of the amalgamated signal to the HYP [149,150,152,153]. The ability of ghrelin to convey messages to the brain via the vagus nerve was investigated shortly after its discovery in an attempt to uncover how ghrelin elicits its central effects despite its predominant gastroenteric system production and poor transport across the BBB [6,18,19,20,28].

Subdiaphragmatic vagotomy, gastric branch vagotomy, and selective destruction of vagal nerve afferents completely eliminate the orexigenic effects that ensue following peripheral ghrelin administration [123,154]. Furthermore, destruction of vagal afferents abolishes the upregulation of NPY transcript and c-Fos expression that traditionally follow peripheral ghrelin administration [123,154]. It is important to note that vagotomised rodents maintain their ability to respond to centrally injected ghrelin [123,154]. Similarly, vagotomised human patients also do not increase their food intake in response to peripheral ghrelin injections [155]. Consistent with this, GHSR transcript is present in vagal afferent cell bodies of the nodose ganglion and GHSR protein is found on axon terminals innervating the gastrointestinal tract [123,156]. Electrophysiological studies demonstrate that in contrast to anorectic peptides, ghrelin suppresses gastric vagal afferent activity at doses known to enhance feeding [123,157].

Closer examination of the vagal afferent associated neural pathways that are engaged following peripheral ghrelin administration led to the realization that projections from the NTS to the ARC of the hypothalamus were required for the observed increases in food intake [152]. Specifically, Date and colleagues [152] found that bilateral midbrain transections, which do not significantly alter feeding or body weight on their own, severs the efferent fibers of the NTS to the ARC and completely abolishes the ability of peripheral ghrelin to induce feeding relative to controls. Again, these transections do not prevent animals from increasing their food intake following central ghrelin administration [152]. The noradrenergic system appears to be intimately involved in mediating the central orexigenic effects initiated by peripheral ghrelin [152]. More specifically, the noradrenergic system influences feeding circuits downstream of the integration of vagal afferent signals within the NTS [152]. This notion is supported by several pieces of evidence. First, the afferents sent to the HYP from the NTS are primarily noradrenergic in nature [152,158]. Second, the enzyme that synthesizes noradrenaline (i.e., dopamine β hydroxylase) is significantly increased in the NTS following peripheral ghrelin administration [152]. Third, bilateral midbrain transections prevent the increase in noradrenaline overflow within the ARC seen following peripheral ghrelin injections [152]. Lastly, eliminating neurons in the hindbrain that express dopamine β hydroxylase is sufficient to block the orexigenic effect of peripheral ghrelin [152]. While these data do not fully demonstrate that noradrenaline exclusively mediates the orexigenic effects of ghrelin as adrenergic cells in the NTS also produce NPY (a potent orexigenic peptide), these data support the idea that peripheral ghrelin exerts some of its orexigenic action through vagal afferents that include the AP and the NTS.

Conversely, there are also reports that suggest that vagal afferents are not required for peripheral ghrelin to stimulate feeding behaviours [159]. Studies conducted by Arnold and colleagues [159], which used a very selective subdiaphragmatic vagal deafferentation method and stringent vetting criteria of rodents that did not meet satisfactory deafferentation, show that intact vagal afferents are not required to increase feeding after intravenous ghrelin. Even after adopting the same vagotomy method as previous studies [154], Arnold and colleagues [159] report that vagotomised animals increase their food intake to the same extent as sham lesioned animals following peripheral ghrelin treatment. This indicates that vagal afferents are not absolutely essential to engage neural orexigenic pathways following peripheral ghrelin administration and suggests that peripheral ghrelin also targets central GHSRs to initiate feeding.

Together these studies demonstrate that peripheral ghrelin likely activates GHSRs within the periphery (i.e., vagal afferents) as well as those found within the brain to induce feeding. It is important to remember that both vagotomy and transection studies undoubtedly affect a plethora of peripheral and central signals that influence feeding behaviours aside from those involved in ghrelin signalling [160,161,162]. Therefore, caution must be taken in interpreting data from these studies and efforts must be made to control for as many confounds as possible in future studies. Transgenic model experiments that specifically allow one to manipulate GHSR positive cells of the vagal afferent following peripheral ghrelin infusion will be valuable in elucidating the role that GHSR positive vagal afferents have in transducing the orexigenic effect of peripheral ghrelin. For example, Designer Receptors Exclusively Activated by Designer Drugs (DREADD)-based experiments could be designed to evaluate feeding behaviours following peripheral administration of ghrelin whilst GHSR positive vagal afferents are inhibited or excited. Alternatively, one could assess peripheral ghrelin induced feeding behaviours in mice that have impaired GHSR function (i.e., knockout or knockdown) specifically in vagal afferents to clarify the importance that GHSR positive vagal afferents have in conveying peripheral ghrelin’s appetite stimulating effects.

### 3.2. Ghrelin and Circumventricular Organs

Circumventricular organs (CVOs) are unique brain regions that do not possess conventional BBBs. As such they are important brain regions involved in the bi-directional transport of molecules between the brain and the circulation. They are endowed with receptors for a variety of molecules and have distinct capillary networks (i.e., fenestrated and curvy) that promote high blood volume but low blood flow, features that facilitate the transfer of molecules into and out of the brain [75,163,164,165]. These specialized characteristics make CVOs abnormally sensitive to circulating substances that are unable to cross the BBB due to their size, charge, chemical profile (i.e., lipophilic), or lack of specialized transport [139,165,166,167,168]. For these reasons CVO are of great interest to those probing the orexigenic capacity and direct central targets of peripheral ghrelin.

Although CVOs more freely allow substances from the blood into the brain, they do so in a highly regulated manner. Recently, specialized ependymal glial-like cells (i.e., tanycytes) that line portions of the ventricles adjacent to CVOs, have been recognized as important for regulating the passage of molecules between the blood, CSF, and the brain [169,170,171,172]. There are as many as eight proposed CVOs (i.e., not all recognized as true CVOs) which belong to one of two main CVO categories: secretory or sensory [167,173,174]. The well recognized and accepted secretory CVOs include the median eminence (ME), neurohypophysis, pineal gland, and the intermediate lobe of the pituitary gland [173,174]. Conversely, the conventional sensory CVOs are the SFO, AP, and the vascular organ of the lamina terminalis [173,174]. The focus here within will solely be on the median eminence, SFO, and the AP as the peripheral action of ghrelin within these regions has been the most thoroughly investigated.

#### 3.2.1. Median Eminence

The ME is situated below the third ventricle at the base of the HYP [175]. It shares its dorsal lateral borders with the ARC [175]. Consistent with its classification as a CVO the ME houses a plexus of fenestrated capillaries and is important for relaying messages between the brain and the rest of the body [175,176,177,178]. The ME, considered a secretory CVO, is uniquely designed to allow hormones produced in the brain to be dumped into the circulation but also, to some extent, permit circulating substances to enter the ME [172]. Tanycyte cell bodies residing along the ventral surface of the third ventricle (i.e., β-tanycytes) have cellular processes that make contact with blood vessels as they make their way through the ventral-dorsal axis of the ME [169,177,178,179]. Originally, tanycytes were thought to liberally move macromolecules bi-directionally between the blood and CSF via transcytotic processes; however, this has been questioned by some [177,180,181]. Accordingly, when administered intravenously, Evans Blue dye (common dye used to examine BBB integrity) is able to pass from the blood into the ME via tanycyte processes but is not transported into the neighbouring ARC or CSF [177]. Conversely, when Evans blue dye is administered into the third ventricle, lateral tanycytes, which border the lateral sides of the third ventricle (i.e., α-tanycytes), transfer the dye into the ARC but not into the ME or any other hypothalamic nucleus [177,181]. These data suggest that the bidirectional transport of molecules from the blood into the ME and neighbouring hypothalamic regions as well as the transfer from the CSF into these nuclei is a complex process regulated by different subtypes of specialized cells (i.e., α and β tanycytes) [172].

The ME has long been a CVO of interest to ghrelin researchers due to its proximity to the ARC, an indispensable brain region for the orexigenic effects elicited by ghrelin [79,80]. As aforementioned, the ARC expresses GHSR mRNA and protein [13,14,17,65,79,80] and exhibits strong c-Fos staining in response to peripheral ghrelin injections [78,125,127,130]. The neurocircuits that transduce ghrelin’s orexigenic signal within this nucleus are also well characterized (Figure 1) [21,22,64,82,83,84,85,89,90,91,92,93,94,95,126]. Despite this, whether ghrelin directly targets the arcuate nucleus has, until recently, been unclear due to disparate data concerning the permeability of the ARC’s BBB [71,126,167,182,183,184,185]. Moreover, although speculated that ghrelin may bypass the BBB by travelling through the ME to the ARC, this idea was deemed improbable due to the general limited diffusion of molecules within the brain and aforementioned gating of transport between the ME and ARC by tanycytes [75,172,177,186,187,188,189]. Fortunately, McGirr and colleagues [133] designed a tool to test the capacity of ghrelin to access central targets, a truncated 18 amino acid form of ghrelin (i.e., fluorescine-ghrelin) conjugated to a fluorescent molecule (i.e., fluorescein isothiocyanate). Fluorescein-ghrelin is capable of stimulating feeding and demonstrates equivalent receptor binding affinity, serum stability, and agonist activity as endogenous acylatedghrelin [133,190]. Interestingly, a positive fluorescent signal is observed only in the ARC and ME following peripheral administration of fluorescein-ghrelin at a dose that mimics those found after an overnight fast in rodents (i.e., 2 fold increase in plasma ghrelin) or just prior to meal initiation in humans [122]. Using a different fluorescently tagged bio-active ghrelin derivative (i.e., Cisbio Bioassays) and multiphoton microscopy techniques, Schaeffer and colleagues [21] found that fluorescently labelled ghrelin travels from the circulation through fenestrated capillaries of the ME into the ARC where it binds to NPY and POMC neurons. In these studies, no other GHSR rich brain regions demonstrate detectable fluorescence [21]. Interestingly, fluorescence differed based on nutritional status, with more positively labelled cells (i.e., higher total and NPY positive neurons but no difference in number of positively labelled POMC neurons) in fasted relative to ad libitum fed mice [21]. These data supports the notion that physiologically relevant doses of peripheral ghrelin directly and exclusively target the ARC. Moreover, these studies highlight the ME as an important region whereby peripheral ghrelin enters the brain. However, because these studies utilized fluorescently labelled truncated analogs of human ghrelin instead of full length tagged ghrelin from the indigenous species in which it was to be tested in, it remains circumstantial evidence. Future experiments, which inject full length fluorescently labelled ghrelin from the host species, are required to make more direct and sound arguments concerning the central targets of peripherally produced ghrelin. Furthermore, an in depth analysis of the capacity of ghrelin to access central targets during different nutritional states (i.e., satiated versus fed) would be valuable given that the ability of ghrelin to enter the HYP appears to be influenced by nutritional status [21].

#### 3.2.2. Subfornical Organ

The SFO is located at the base of the fornix dorsal to the lamina terminalis and posteriorly extends into the third ventricle [75,173,191]. Neurons within the SFO directly project to various nuclei of the HYP (i.e., ARC, PVN, LH, supraoptic nucleus, lateral preoptic area) in addition to the infralimbic cortex, bed nucleus of the stria terminalis, zona inceta, and brain stem [191,192,193,194,195,196,197]. Importantly, the SFO expresses receptors for and has the capacity to respond to many circulating substances involved in energy balance, including ghrelin [130,195,198,199,200]. c-Fos data following peripheral ghrelin administration is inconsistent within the SFO. Clear increases have been reported by some while others fail to detect differences in ghrelin versus vehicle treated rats [130] but see [128]. Physiologically relevant doses of ghrelin stimulate specific subsets of neurons within the SFO that are distinct from neurons which are activated by the anorectic peptide amylin [200]. This activation is concentration dependent and blocked by GHSR antagonists suggesting that ghrelin levels must surpass a certain threshold before GHSR activation is sufficient to stimulate SFO neurons [200]. These studies are valuable in that they identify ghrelin responsive cells within the SFO; however, they do not address whether endogenous ghrelin definitively targets the SFO to activate pathways involved in feeding and/or to gain access to the CNS. A recent study that peripherally injected a fluorescent labelled ghrelin analog suggests that the SFO is not one of the brain regions that peripheral ghrelin directly targets to elicit its effects [122].

#### 3.2.3. Area Postrema

The AP is situated at the intersection of the medulla and the spinal cord at the ventroposterior boundary of the fourth ventricle [173]. The AP sends projections to a number of brain regions including the NTS, reticular formation, lateral parabrachial nucleus, dorsal motor nucleus of the vagus, as well as to tegmental nuclei within the brainstem [201,202,203]. Furthermore, it receives reciprocal inputs from the NTS and lateral parabrachial nucleus in addition to those from the HYP (i.e., PVN and DMH) [201,202,203]. Consistent with its classification as a CVO, the AP is highly vascularised (i.e., by vessels not protected by the BBB), contains neurons that express a wide variety of receptors, and is well situated to receive circulating metabolic signals [173,198].

Perhaps not surprising given the ability of ghrelin to modulate energy balance processes, GHSR transcript is highly expressed within the AP and neighbouring brain regions innervated by the AP (i.e., NTS, and the DMNV) [17]. Consistent with this, GHSR-green fluorescent protein reporter mice also demonstrate high expression of the GHSR protein within the dorsal vagal complex (DVC) (i.e., AP, NTS, DMNV), particularly within the AP [14]. Correspondingly, injection of ghrelin into either the fourth ventricle or into the DVC induces a hyperphagic response [204]. Electrophysiological experiments confirm that the AP houses neurons (~40%) that are responsive to physiological concentrations of ghrelin [205]. Similar to neurons within the SFO, the response of neurons within the AP is concentration dependent and blocked by application of a GHSR antagonist [205]. Peripheral administration of ghrelin or ghrelin analogs induce strong c-Fos immunoreactivity in all brain regions of the DVC [128,129,130,206]. Likewise, the AP is the only brain region, aside from the ARC, that demonstrates a positive signal following peripheral administration of a fluorescently tagged bioactive ghrelin analog (although at very high dose) [122]. Together these data suggest that peripheral ghrelin may target the AP and associated feeding related brain regions that it innervates (e.g., NTS and DMNV) to modulate food intake [122]. In support of this, lesion of the AP completely prevents increases in c-Fos expression within the other two DVC brain regions (i.e., NTS and DMNV) following peripheral ghrelin administration [129]. Further supporting that the AP is an important relay station for the orexigenic effects elicited by peripheral ghrelin, AP lesioned animals do not increase their food intake following chronic peripheral injections of ghrelin relative to controls [207]. It has been suggested that projections from the DVC to the HYP are responsible for the orexigenic effects elicited by ghrelin in the brainstem [149]; however, some argue that forebrain and hindbrain feeding circuits are independent [152,208]. Either way, the AP is an important nucleus whereby peripheral ghrelin conveys orexigenic signals.

## 4. The Role of Central Ghrelin in Stimulating Feeding

### 4.1. Central c-Fos Studies

Irrespective of current evidence suggesting minor passage of ghrelin from the periphery into the CNS, it is indisputable that the central ghrelin system nonetheless plays an important role in regulating feeding behaviours. Consistent with this, GHSRs are widely expressed within many distinct feeding related brain regions such as the HYP, HIP, amygdala, VTA and the caudal brainstem [11,13,14,17,100]. Furthermore, biotin-labeled ghrelin experiments demonstrate that ghrelin efficiently binds GHSRs in GHSR rich brain regions [1,3,22]. Accordingly, both microinfusion of ghrelin into GHSR rich brain regions and intracerebroventricular (ICV) administration of ghrelin encourages motivated behaviours to acquire and consume food [1,6,9,10,67,119,122,209]. Not surprisingly, c-Fos expression patterns following ICV ghrelin administration closely resemble the distribution pattern of GHSRs, with dense expression in brain regions known to influence feeding (Figure 3) [6,14,17,76,209]. Lastly, it has been recently demonstrated that mice, which lack functional GHSRs specifically in neurons, do not increase the amount they consume following peripheral ghrelin administration [132]. Given the aforementioned evidence, it is clear that activation of central GHSRs encourages feeding behaviours.

### 4.2. Cerebrospinal Fluid Ghrelin

The clear induction of GHSR dependent behavioural effects (e.g., increased motivation to obtain and consume foods) following ICV administration of ghrelin prompted questions concerning the origin and physiological role of CSF ghrelin [1,10,119]. Although central CSF ghrelin levels have not been quantified in rodents due to technical difficulties (i.e., large amount of sample is needed for current assays), experiments conducted with human or sheep subjects report approximately 10 to 1000 fold lower levels of ghrelin in the CSF relative to those found in blood [210,211]. In sheep, peripheral injections of ghrelin that quickly raise circulating levels 10 fold only cause a doubling of CSF acylated ghrelin levels (levels peaked 50 min after ghrelin injection) suggesting that although peripherally injected ghrelin appears to be able to cross the BBB into the CSF its transport is limited and slow [210]. Moreover, it is not inconceivable that CSF ghrelin may also originate from central sources; however, this notion remains controversial and will be discussed elsewhere in this manuscript [22,23,24,25]. Given the few studies that have investigated CSF acylated ghrelin levels in conjunction with those that have examined the concentration of acylated ghrelin required to induce reliable activation of GHSRs, it appears that CSF acylated ghrelin concentrations are likely inadequate to initiate GHSR dependent processes [49,75,205,212,213]. Nonetheless, since diurnal CSF ghrelin concentrations or those that accompany food restriction and/or fasting conditions have not been characterized, the capacity of CSF acylated ghrelin to initiate GHSR dependent processes should not be completely discounted. Furthermore, circulating acylated ghrelin is rapidly converted into des-acyl ghrelin by esterases present in the circulation and thus CSF acylated ghrelin levels may be underestimated [59]. Therefore, enhancing the sensitivity of current ghrelin quantification assays and developing more efficient sampling methods that maximize recovery and mitigate degradation of acylated ghrelin may make CSF ghrelin detection possible in rodents and more accurate in other model species. Fortunately, compounds able to reliably prevent hydrolytic degradation of acylated ghrelin into des-acyl ghrelin in biological samples have recently been identified [59]. These ameliorations will go a long way in determining whether endogenous CSF ghrelin levels relevantly influence GHSR dependent behaviours such as feeding.

Regardless, it appears that CSF ghrelin is capable of accessing various GHSR rich feeding related brain regions as ICV injection of fluorescein-ghrelin results in a fluorescence distribution pattern that largely overlaps with past reports examining GHSR mRNA expression (Figure 3) [13,17,190,210]. The fluorescein-ghrelin signal is attenuated by ICV administration of unlabeled ghrelin in wild-type (WT) mice and is not observable in any nuclei of GHSR KOs suggesting that fluorescein-ghrelin specifically binds to GHSRs [190]. The highest number of fluorescently labelled cells and brightest signal is found in the HYP; however, positively labelled neurons are also found in many extrahypothalamic sites (e.g., HIP, dorsal raphe nucleus, VTA, laterodorsal tegmental nucleus) [190]. Interestingly, fluorescein-immunoreactive signal is ubiquitous in ependymal like cells that border the ventricular system (i.e., lateral, third and fourth ventricles, aqueduct of Sylvius, and the central canal) in both WT and GHSR null mice [190]. Moreover, bright signals are detected in the cell bodies and far reaching ME cellular projections of tanycytes that line the bottom of the third ventricle in both genotypes [190]. These experiments highlight plausible GHSR independent processes by which CSF ghrelin crosses the CSF brain barrier to access brain regions that house GHSRs [190]. Consistent with this, others have reported tanycyte dependent plastic changes in the permeability of the blood CSF interface in response to metabolic factors [169,171]. Although the GHSR independent mechanism by which ependymal cells internalize and pass CSF ghrelin to brain parenchyma is currently unclear, these studies suggest that CSF ghrelin can reach widespread GHSR positive BBB privileged brain regions [190]. Advances in the collection and detection of acylated ghrelin within the CSF will clarify whether CSF ghrelin concentrations similarly fluctuate with feeding status and are adequate to bind and activate GHSRs. Positive results in this regard, would suggest that ghrelin uses the CSF and ventricular system to reach its widespread CNS targets. It is important to note that according to our knowledge no one has investigated whether GOAT is expressed in CSF. Therefore the possibility that des acyl ghrelin is acylated in the CSF and then distributed to the aforementioned BBB protected GHSR rich brain regions cannot be dismissed.

### 4.3. Central Ghrelin Synthesis

A hotly debated question amongst ghrelin researchers is whether or not ghrelin is synthesized within the brain. Although, the contention is mainly fueled by conflicting evidence surrounding the subject, the significance of central ghrelin synthesis in and of itself makes it an important and intriguing topic. This is especially true since, as discussed previously, ghrelin has been predominantly viewed to be produced exclusively in the periphery and with the exception of the ME and to a lesser extent the AP does not appear to appreciatively cross or avoid (i.e., enter the brain via circumventricular organs) the BBB to access central GHSRs [18,19,122]. Therefore, central ghrelin synthesis and secretion, if proven to occur, would go a long way in reconciling why GHSRs are found in numerous BBB privileged feeding related regions that are seemingly inaccessible to peripheral ghrelin.

#### 4.3.1. Support for the Central Synthesis of Ghrelin

There is a substantial amount of evidence suggesting that ghrelin is synthesized within the brain (Table 1). To begin, many independent groups have detected ghrelin mRNA within the brain (most commonly within the HYP) [20,22,24,56,214,215,216,217,218,219]. Second, the hypothalamic expression of ghrelin and GOAT mRNA fluctuate with feeding status (i.e., upregulated following a 48 h fast in rats) [56]. Although hypothalamic ghrelin mRNA is not upregulated in food restricted animals, the transcript for GOAT is suggesting that elevations in ghrelin signaling may be due to enhanced activation of ghrelin rather than upregulation of the peptide itself [56]. Further alluding to the importance of central ghrelin synthesis and/or activation, chronic central GOAT knockdown (via ICV infused amorpholino antisense oligonucleotides) significantly decreases weight gain of rats fed a high fat diet [56]. Third, ghrelin protein has also been detected within the brain by many independent groups [20,22,24,216,220,221,222,223,224,225]. Not surprisingly, these ghrelin producing cells most commonly are described within hypothalamic nuclei but some extra-hypothalamic ghrelin containing processes have been reported [20,22,24,215,219,223,224]. Studies demonstrating positive central ghrelin immunoreactivity have been conducted in rats [20,22,24,60,215,219,220,221,223], mice [22], and humans [224] and have used antibodies targeting both des-acylated [22,220,221,223,224] and acylated ghrelin forms [24,215,219]. Oddly, although most of these studies report expression within the ventral portion of the HYP, the precise cellular (i.e., specific neuron types) and sub-cellular (i.e., soma versus axons/terminals) location of ghrelin differs greatly [20,22,24,60,215,219,223,224]. Since a majority of these antibodies were validated (i.e., ghrelin KO animals, pre-incubation of anti-sera in an excess of ghrelin, etc.), the discrepancy in the distribution of ghrelin immunoreactivity appears to reflect differences in species of study and specific ghrelin epitope targeted rather than poor specificity. Nonetheless, many still harness skepticism concerning the specificity of currently available antibodies as well as the validity of associated evidence supporting central ghrelin synthesis [23,25,218]. Even so, the fact that many independent groups have utilized unique antibodies targeting different ghrelin epitopes and all report ghrelin-immunoreactivity specifically within the HYP strongly supports the notion that ghrelin is synthesized centrally. Moreover, Kageyama and colleagues [225] provided support for ghrelin’s central synthesis without relying on anti-ghrelin antibodies by creating transgenic mice that express enhanced green fluorescent protein (EGFP) in cells that produce ghrelin (i.e., inserted the gene for EGFP in the regulatory region of the ghrelin gene). As expected, these mice express both EGFP and ghrelin mRNA in the stomach and likewise show immunoreactivity for ghrelin and GFP proteins within this region [225]. Importantly, ghrelin and EGFP transcripts and corresponding proteins are detectable within the HYP of these mice, supporting the fact that ghrelin producing cells exist within this region [225]. Lastly, radioimmunoassays of push-pull perfusion perfusates also support ghrelin’s central production and release [226]. Ghrelin levels in the ARC were shown to rise almost 2 fold relative to baseline following intra-ARC infusion of potassium ions (i.e., encourages depolarization) [226]. This effect was found to be extremely specific as animals with off-target probes did not demonstrate enhanced ghrelin levels [226]. Although one cannot completely exclude the putative contribution of peripheral ghrelin, the steep enhancement of ghrelin evoked by the potassium ions strongly suggests local terminal vesicle release within the ARC. Collectively, the aforementioned data gathered using a multitude of different techniques (i.e., in situ hybridization, real-time polymerase chain reaction (RT PCR), immunohistochemistry, transgenics, push-pull perfusion, radioimmunoassays) make a strong case for the central synthesis and release of ghrelin, at least within the medio-basal hypothalamic region.

#### 4.3.2. Evidence Opposing the Central Synthesis of Ghrelin

In a similar fashion, a number of studies have provided data that refutes the notion of ghrelin’s central synthesis (Table 1) [23,25,218]. For example, transgenic ghrelin deficient mice (*ghrl^−/−^*), which were engineered to express a *lacZ* reporter gene in the place of the functional ghrelin gene, demonstrate evident β-galactosidase staining within the stomach and intestine but no positive staining within the HYP [218]. Furthermore, WT and ghrelin deficient mice show negligible and indistinguishable hypothalamic ghrelin immunoreactivity despite having very different stomach ghrelin immunoreactivity profiles (i.e., strong signal in WTs and absence of signal in *ghrl^−/−^s*) [218]. Although Wortley and colleagues were able to detect low levels of ghrelin mRNA within WT animals, their aforementioned inability to detect acylated ghrelin or to observe β-galactosidase staining within the HYP contradict previous claims that ghrelin is found and/or is synthesized centrally. Consistent with this, studies run with two separate transgenic ghrelin reporter mice, in which humanized *Renilla reniformis* GFP expression was driven by different lengths of the ghrelin promotor, failed to detect GFP fluorescence in the brain but demonstrated clear GFP expression in the stomach [25]. It is important to note that within these two transgenic ghrelin reporter mice lines no signal was observed in other tissues known to express ghrelin to a lesser extent (i.e., pancreas, intestine, kidney) [25,28,227,228]. For instance, although in situ-produced ghrelin has been established as an important endocrine signal for the development of the pancreas and the regulation of insulin release [229,230], ghrelin reporter systems fail to detect a signal within this organ [25]. Therefore, although these reporter lines appear to be useful for detecting tissues that produce high levels of ghrelin, their utility in detecting regions that synthesize small amounts of ghrelin, as suspected within the HYP, may be limited. Nonetheless, since ghrelin expression may vary during development, examination of these transgenic lines during times when ghrelin is supposedly expressed most strongly may clarify whether ghrelin reporter lines support the extragastric production of ghrelin [231]. Additionally, Sakata and colleagues [25] failed to detect ghrelin immunoreactivity or ghrelin mRNA (via in situ hybridization) within the brain of WT or transgenic reporter mice. Their inability to detect ghrelin mRNA or protein within the brain argues against the central synthesis of ghrelin; however, it may also be that the quantity of ghrelin produced within the HYP does not surpass inherent detection thresholds. Lastly, due to concerns over the specificity and type of ghrelin (e.g., des-acyl ghrelin, preproghrelin) targeted by antibodies in immunohistochemical studies supporting central ghrelin synthesis, in addition to evidence of the contrary [25,218], Furness and colleagues [23] conducted a comprehensive analysis of ghrelin protein expression in the rodent brain. They report strong signals from the stomach but no specific immunoreactivity for either ghrelin or des-acyl ghrelin in the CNS (e.g., HYP, medulla oblongata, and spinal cord) using four different antibodies (three targeting acylated ghrelin and one targeting des-acyl ghrelin) [23]. The authors argue that the aforementioned histochemical studies that demonstrate ghrelin immunoreactivity in the HYP are likely detecting ghrelin precursors or compounds with sufficient similarities to the ghrelin peptide [23]. Collectively, the data presented above represents the most persuasive evidence contesting the central synthesis of ghrelin.

#### 4.3.3. Future Avenues to Ease the Central Synthesis of Ghrelin Debate

Although strong evidence exists for the central synthesis of ghrelin, equally convincing experiments have been conducted and lend support to the opposite conclusion. It is evident that more work is needed to clarify whether or not ghrelin is centrally synthesized in the brain. The ability to detect ghrelin or ghrelin reporter immunoreactivity within the brain is one of the biggest points of contention between those that believe that ghrelin is synthesized centrally and those that oppose it.

All available anti-ghrelin antibodies (including those that have detected central ghrelin and those that have not) need to be characterized (e.g., via immunohistochemical studies) in both ghrelin deficient and WT rodents. More specifically, this characterization should involve an in depth analysis of all extra-gastric tissues reported to have the capacity to synthesize ghrelin and efforts should be made to include amplification steps to maximize the detection of ghrelin in organs that may weakly produce it (e.g., pancreas, intestine, kidney, brain). These experiments will not only be useful in identifying which antibodies are specific but will also highlight published works that may have used non-specific and/or poor quality antibodies. Correspondingly, this will facilitate identification of data that should be interpreted with caution and will highlight the best antibodies to use for the future detection of ghrelin. Measuring ghrelin levels (e.g., microdialysis) directly within the HYP with verified antibodies would provide extremely important information pertaining to the validity of the central synthesis of ghrelin. Unfortunately, quantifying ghrelin levels via microdialysis is not a common practice, primarily due to low recovery (i.e., approximately 30% of actual levels) [232]. However, as ghrelin and GOAT transcript levels within the HYP are increased following food restriction, measuring ghrelin levels in hypothalamic regions during a time of energy insufficiency, may heighten the concentration of ghrelin to a detectable level [56]. Likewise, early addition of alkyl fluorophosphonate compounds, which slow the degradation of acylated ghrelin (through the inhibition of enzymes that cleave the ester bond attaching the octonyl moiety to the rest of the ghrelin peptide), to sample perfusates should also ameliorate detection thresholds [59]. Alternatively, high-performance liquid chromatography-mass spectroscopy techniques could be exploited to assess central acylated ghrelin levels. It is important to note, that these experiments would have to be conducted in nuclei thought to be inaccessible to peripheral ghrelin to confirm ghrelin’s central synthesis.

Lastly, current ghrelin reporter transgenic lines largely fail to express reporter genes in extra-gastral tissues known to be able to produce and secrete ghrelin and/or demonstrate weak signals relative to background [25,218,225]. Accordingly, generating transgenic ghrelin reporter mice that more accurately express reporter genes in tissues and cell-types that naturally produce ghrelin (by transfecting bacterial artificial chromosomes that contain larger fragments up and down stream of the ghrelin gene) is of prime importance. If accomplished, these transgenic ghrelin reporter lines will not only be a valuable asset in determining if ghrelin is centrally synthesized but will facilitate the study of ghrelin cell physiology in general.

## 5. GHSR Activity Independent of Ghrelin

Although peripheral ghrelin clearly modulates feeding behaviours via various means (e.g., vagal afferents, CVO entry and activation of central GHSRs) and centrally synthesised ghrelin may also contribute, it has become apparent that GHSRs may also convey feeding related messages independent of ghrelin [6,9,10,22,123,152,154,225,233]. Accordingly, the GHSR demonstrates very high constitutive activity (i.e., initiates downstream signaling events in the absence of suitable ligands) and interacts with a plethora of other GPCR in addition to itself [212,233,234,235,236,237]. Consistent with this, when challenged (e.g., feeding regimes, stressed) ghrelin KO animals have different feeding behaviours and phenotypes relative to animals that lack a functional ghrelin receptor (i.e., GHSR KO or GHSR null rodents) [1,38,238,239,240,241]. Ghrelin independent activation of GHSRs may help rectify the apparent discontinuity between the ubiquitous expression of GHSRs within BBB protected feeding related brain regions and the limited accessibility of ghrelin to these regions. The following sections will describe the putative roles that GHSR constitutive activity and dimerization play in modulating feeding behaviours.

### 5.1. GHSR Constitutive Activity

A vast majority of GPCRs are constitutively actively to some extent; however, the degree of constitutive activity displayed by the GHSR is uniquely high [212]. It has been reported that the GHSR, on its own, can signal at about half the capacity as that elicited following ghrelin binding [212,242]. This estimation has been substantiated using a number of different assays and measures (e.g., inositol phosphate turnover and cyclic adenosine monophosphate (cAMP) response element-induced transcriptional activity) [212,242]. Although it has been argued that the reported constitutive activity of the GHSR is overinflated due to assay dependent parameters (e.g., unnaturally high levels of expression in heterologous systems or intrinsic modulators) [233,235,243], studies that limit many of these confounds confirm the high constitutive activity of GHSRs and report that it is an intrinsic property of the receptor [244].

Modulation of this high constitutive activity has corresponding ramifications on GHSR dependant biological processes such as feeding and energy balance [242,245,246,247]. A study examining a genetic mutation in the GHSR gene, shared amongst individuals with familial short stature, was the first to examine the in vivo consequences of altered GHSR constitutive activity [246]; however, the importance of ligand independent GHSR basal activity in regulating appetite had been previously suggested [242]. The in vivo use of [d-Arg^1^, d-Phe^5^, d-Trp^7,9^, Leu^11^]-substance P, a weak antagonist but potent inverse agonist (counteracts downstream signaling cascades induced by GHSR activation) of the GHSR, clarified the necessity of basal GHSR activity in the regulation of feeding and energy balance [212,247]. Accordingly, central blockage of constitutive activity, via chronic ICV administration of [d-Arg^1^, d-Phe^5^, d-Trp^7,9^, Leu^11^]-substance P, is sufficient to decrease both food intake and body weight as well as the hypothalamic expression of NPY and uncoupling protein 2 (UCP-2) compared to controls [247]. The alteration in NPY and UCP-2 expression is suspected to be mediated by GHSR dependent cAMP response element-binding protein (CREB) phosphorylation, as CREB phosphorylation is increased by ghrelin and decreased by [d-Arg^1^, d-Phe^5^, d-Trp^7,9^, Leu^11^]-substance P in hypothalamic cell lines [247]. Likewise, ICV administration of another potent GHSR inverse agonist (i.e., K-(d-1-Nal)-FwLL-NH_2_), known to counteract GHSR constitutive activity, severely decreases short term food intake (almost 5-fold) relative to control animals [245]. Further corroborating a role for GHSR constitutive activity in regulating feeding, presynaptic constitutive GHSR activity impairs calcium currents within rodent hypothalamic ARC neurons and reduces GABA release from these explants [248]. Lastly, alluding to a means for the regulation of constitutive activity, GHSRs are upregulated in the HYP during times of energy insufficiency (i.e., during fast or chronic food restriction) when food procuring behaviours are needed [56,248,249,250,251]. Collectively these data strongly suggest that high GHSR constitutive activity is important for regulating energy balance (i.e., feeding and metabolism) and highlight the HYP as at least one location whereby this activity engages signaling events that promote orexigenic neural circuits [247]. Furthermore, it highlights the efficacy that inverse agonists have with respect to curbing food consumption and thus their therapeutic potential in combating excessive feeding [242,245].

### 5.2. GHSR Promiscuity

Heterodimerization between GPCRs is an increasingly apparent phenomenon that is attracting a lot of attention given the functional and physiological implications associated with it. It not only provides another means by which central systems can influence one another but also alters the way in which we interpret data. Accordingly, heterodimerization between GPCRs can alter the strength and/or nature of downstream signalling cascades transduced by corresponding monomeric GPCRs [251,252]. These changes are most frequently a consequence of altered ligand binding, G-protein selection, or modified receptor trafficking to or from the cell membrane (i.e., receptor internalization) [253,254]. Importantly, the realization of the existence and prevalence of heterodimer formation may partially explain the distribution pattern and function of receptors thought to be inaccessible to their respective ligands, particularly the GHSR [233,255]. Accordingly, GHSR-1as (growth hormone secretagogue receptors type 1a), which up until this point have been referred to simply as GHSRs, have the capacity to form heterodimers with many GPCRs: most notably GHSR-1bs, melanocortin 3 receptors (MC3-Rs), serotonin 2C receptors (5-HT_2C_), and dopamine receptors 1 (DRD1) and 2 (DRD2) [235,236,237,238,256,257,258,259,260,261]. These heterodimers have been characterized in vitro and are just starting to be examined in ex vivo and in vivo studies [235,236,237,238,257,259,261]. Evidence supporting the formation of these heterodimers and their potential role in regulating feeding will be discussed subsequently (Table 2). It is important to note that to date the physiological in vivo evidence that may be used to support theories of heterodimer formation in vivo may simply be indicative of an interaction between two systems. More advance techniques and rigorous testing are needed to confirm the location and prevalence of heterodimers in vivo and the extent to which they may influence signaling cascades and ultimately behaviour.

As stated throughout this paper, activation of central GHSRs unequivocally enhances both the motivation and consumption of food [6,10,67,70,119,122,209]. The α G-protein subunit (i.e., α_q_, α_s_, or α_i/o_) that GHSRs associate with and the corresponding signaling cascades that are initiated following ghrelin binding are diverse and are influenced by a multitude of factors (e.g., tissue, cell type, heterodimer partner) [4,262,263,264]. Most commonly, GHSRs couple with Gα_q_ and upon activation elicit increases in intracellular calcium via phospholipase C/inositol triphosphate (PLC/IP_3_) signalling cascades [4,13,261,263,264]. Particularly, within the HYP, this rise in intracellular calcium is important for the mobilization of calcium/calmodulin-dependent protein kinase II (CaMKII) and subsequent activation of AMPK signalling cascades that promote orexigenic responses [68,265,266,267,268]. GHSR Gα_s_ dependent stimulation of AMPK via cyclic adenosine monophosphate/protein kinase A (cAMP/PKA) signaling cascades have also been reported [222,268]. Ghrelin is likewise capable of stimulating GHSR dependent signalling pathways that are sensitive to pertussis toxin indicating that the GHSR can also associated with Gα_i/o_ [269,270]. It is clear that the GHSR, like most GPCRs, is extremely versatile in that it can transduce signals through various signalling cascades depending on its microenvironment [263].

### 5.3. GHSR Dimers

Interestingly, although homodimerization between GHSR-1as does not appear to significantly alter ligand binding, G-protein selection, or constitutive activity relative to GHSR-1a monomers, heterodimerization between GHSR-1a and its truncated splice variant, GHSR-1b, has been shown to greatly impact these processes [235,257,280]. The GHSR-1b, which lacks transmembrane domains 6 and 7, does not possess signaling capabilities as a monomeric unit; although, evidence suggests that it may be an integral unit of other functional GPCR oligomers (i.e., GHSR-1a/GHSR-1b/DRD1) [258,261]. Accordingly, heterodimer studies in lipid discs have demonstrated that the GHSR-1a/GHSR-1b heterodimer retains the ability to bind GHSR-1a agonists but loses the capacity to stimulate associated signaling proteins (i.e., Gα_q_ protein or arrestins), ultimately stranding the heterodimer in a non-active state [257]. Consequentially, the heterodimer does not possess the typical high constitutive activity intrinsic to GHSR-1as [257]. This data supports earlier work done in HEK 293 cells demonstrating that co-expression of GHSR-1a and GHSR-1b leads to heterodimer formation, normal ghrelin binding but reduced GHSR-1a constitutive activity and cell surface expression [280]. More recent analysis of this heterodimer in HEK 293 cells shows that the cell surface expression of the heterodimer is actually dependent on the ratio of GHSR-1b versus GHSR-1a (i.e., ratio can be manipulated by co-transfecting cells with disparate quantities of receptor cDNA) [258]. Accordingly, a low ratio of GHSR-1b relative to GHSR-1a promotes cell surface expression of the heterodimer and enhances Gα_i/o_ dependent signaling cascades in response to ghrelin administration [258]. Conversely, a high ratio of GHSR-1b relative to GHSR-1a decreases cell surface expression of the heterodimer and dampens GHSR-1a signalling following ghrelin administration [258]. Interestingly, these findings were substantiated in striatal and hippocampal primary cultures which naturally express high and low GHSR-1b and GHSR-1a ratios respectively [258]. As expected, artificially raising the expression of GHSR-1b in striatal (i.e., naturally high GHSR-1b to GHSR-1a expression ratio) and hippocampal (very low inherent GHSR-1b/GHSR-1a expression ratio) cultures decreased and increased the signaling efficiency of ghrelin respectively [258]. These studies suggest a means by which neurons that express these receptors may self-regulate GHSR-1a signalling. Likewise, it was discovered that although this heterodimer preferentially signals via Gα_i/o_ dependent signaling cascades in HEK 293 cells, in both primary cultures it favourably couples to Gα_s_ to drive cAMP production [258]. The physiological roles of this heterodimer and magnitude of its importance in modulating behaviours, especially feeding, have yet to be thoroughly explored; however, given the signaling pathways that the heterodimer initiates in neurons (primarily Gα_s_), the co-expression of GHSR-1a and GHSR-1b in feeding related brain regions (i.e., hippocampus and striatum), and that GHSR-1b has recently been found to play an important role in a functional GPCR oligomer (i.e., GHSR-1a/GHSR-1b/DRD1) (will be discussed subsequently), it is evident that its influence requires further investigation (Table 2).

### 5.4. GHSR and Dopamine Receptor Heterodimers

The investigation of a putative heterodimer pairing between GHSRs, DRD1s, and DRD2s was inspired by commonalities in the localization of their mRNA, similarities in signaling cascades, and the realization that these systems modulate some of the same physiological processes [13,236,256,281,282,283,284,285,286]. In general, stimulation of DRD1s induces cAMP/PKA signalling cascades via Gα_s_ dependent processes; whereas, DRD2 activation conversely activates Gα_i_ coupled proteins to antagonize cAMP production and PKA activity [284,287,288,289,290].

Neurons within the HIP, VTA, and substantia nigra, co-express GHSR and DRD1 receptors [234]. Furthermore, these receptors co-localize in the same sub-cellular location, immuno-precipitate together, and give off a reliable positive bioluminescence resonance energy transfer (BRET) signal solely in the presence of ligands (i.e., dopamine and ghrelin) [234]. In vitro studies conducted in HEK 293 cells co-transfected with both DRD1s and GHSRs show that dopamine and ghrelin synergistically enhance cAMP accumulation above the modest stimulation of dopamine and the negligible induction of ghrelin alone [234]. It appears that the potentiation of cAMP accumulation (following co-administration of ghrelin and dopamine) involves the exchange of GHSR associated Gα_q_ to Gα_i/o,_, the consequential release of accompanying βγ subunits and the final potentiation of dopamine Gα_s_ mediated cAMP accumulation via the engagement of adenylyl cyclase [234]. Interestingly, others suggest that GHSR-1b is also important for the association of GHSR-1a with DRD1 at least within striatal primary cultures [258]. Consistent with this, treatment of striatal primary cultures with DRD1 antagonists prevents the traditional increase in cAMP levels seen following ghrelin administration in GHSR-1a/GHSR-1b dimer experiments [258]. Collectively, these data provide evidence of a collaborative interaction between GHSRs (i.e., both GHSR-1a and GHSR-1b) and the DRD1 (Table 2).

Recently, the functional and physiological role of the GHSR/DRD1 heterodimer within the HIP was explored [256]. Interestingly, DRD1 agonists significantly enhance the formation of GHSR/DRD1 heterodimers within hippocampal neurons [256]. DRD1 and GHSR agonists enhance Ca^2+^ in WT neurons that express the heterodimer but are unable to elicit this effect in GHSR antagonist treated and GHSR KO hippocampal neurons suggesting that the heterodimer is required for Ca^2+^ mobilization [256]. The rise in intracellular calcium is elicited by PLC/IP_3_ signalling cascades and is dependent on the association of DRD1 with Gα_q_ rather than its usual Gα_s_ partner [256]. Most intriguingly, signaling through the heterodimer was deemed essential for enhancing *N*-methyl-d-aspartate (NMDA) currents integral to the induction of long term potentiation and stimulation of synaptic plasticity within the HIP, processes important for hippocampal behaviour and memory [256]. Accordingly, GHSR inactivation, via genetic and pharmacological means, completely attenuates DRD1 regulated hippocampal behaviour and memory (i.e., dopamine induced interference of pre-pulse inhibition and consolidation of contextual fear conditioning) supporting an important physiological role for the GHSR/DRD1 heterodimer [256].

The implications of GHSR/DRD1 heterodimers in relation to feeding has yet to be directly explored; however, there are some findings, in addition to the aforementioned evidence of its capacity to influence behaviour within the HIP that suggest it may likewise play a role in regulating feeding [256]. First, GHSR and DRD1 transcripts are both highly expressed in feeding related regions such as the HYP and mesolimbic dopamine system [13,258,281,290]. Second, modulation of GHSR activity influences behaviours regulated by the dopamine system [1,101,113,118,119,282,291]. Third, like alteration of GHSRs, selective modulation of DRD1s influences food intake (although in the opposite direction) [292,293,294,295]. Lastly, the ability of ghrelin to modulate food reward behaviours relies on functional DRD1s [116]. Together these data support a putative role for GHSR/DRD1 heterodimers in the regulation of feeding behaviours and highlight the merit of its investigation.

Similarly, several pieces of evidence suggest that GHSRs can also form heterodimers with DRD2s. Accordingly, although DRD2 and GHSR mRNA and protein coexist within neurons of the HIP, striatum, and HYP, these receptors co-localize most strongly within the HYP [235]. Consistent with this, fluorescence resonance energy transfer microscopy experiments report GHSR-1a/DRD2 heterodimers in hypothalamic preparations of WT but not GHSR KO animals [235]. Moreover, molecular studies also support the formation of this heterodimer as DRD2 agonists increase intracellular calcium levels in HEK 293 cells which co-express both GHSR-1as and DRD2s but fail to in cells transfected with either receptor independently or in cells pre-treated with either DRD2 (i.e., raclopride) or GHSR antagonists (i.e., JMV 2959) [235]. In these cells, allosteric interactions between GHSRs and DRD2s have been deemed essential for the mobilization of intracellular Ca^2+^ in response to bath applications of dopamine. This effect relies on the heterodimer coupling to Gα_i_, release of Gβγ subunits and subsequent induction of PLC/IP_3_ signalling cascades (mediated by the βγ subunits) [235]. It is also independent of GHSR constitutive activity as attenuating GHSR constitutive activity (via point mutations of GHSR or introduction of Gα_q_ siRNA) does not significantly blunt the calcium response [235]. Consistent with the existence of GHSR-1a/DRD2 heterodimers, pre-treatment of cells with either GHSR-1a or DRD2 agonists reduces Ca^2+^ release induced by subsequent dopamine or ghrelin treatment respectively (i.e., cross desensitization) [235].

The fact that activation or suppression of DRD2, like modulation of GHSRs, influences feeding behaviours in addition to evidence of their structural and functional interaction in vitro led to investigations concerning the physiological relevance of this heterodimer in vivo [235,295,296]. Accordingly, WT mice peripherally injected with cabergoline, a DRD2 agonist known to suppress appetite, reliably decrease their food intake relative to controls; whereas, cabergoline has absolutely no effect on the food consumption of GHSR KO mice [235]. Furthermore, pre-treatment of WT animals with JMV-2959, a GHSR antagonist, prevents the anticipated decrease in feeding following cabergoline injections [235]. Interestingly, this effect is ghrelin independent as both ghrelin WT and KO animals decrease their food intake in response to cabergoline [235]. Therefore, the appetite suppressant ability of DRD2 agonists rely on GHSR-1as but not on the presence of ghrelin.

Collectively, these experiments not only support the existence of GHSR/DRD2 heterodimers but provide insight into the heterodimer’s downstream signalling pathways (i.e., binding of GHSRs to DRD2s allosterically alters DRD2 canonical signaling transduction leading to increased Ca^2+^ mobilization) (Table 2). Unfortunately, the aforementioned studies have primarily focused on the impact that this heterodimer has on canonical DRD2 signaling pathways and behaviours. How this heterodimer influencing signaling through GHSRs and the behavioural consequences of modulating normal GHSR signalling has been largely ignored. Similar studies that probe the relevance of this heterodimer in modulating GHSR signaling and associated behaviours would be extremely valuable. Nonetheless, these experiments highlight that GHSR/DRD2 heterodimers have the capacity to alter ghrelin and dopamine system signaling cascades and ultimately behaviours, particularly those responsible for the regulation of feeding. They also lend support to the notion that GHSRs situated in brain regions considered to be inaccessible to ghrelin can still exert ligand independent control over important behaviours like feeding.

### 5.5. GHSR/5-Hydroxytryptamine_2C_ Heterodimers

The serotonin system has garnered a tremendous amount of research in recent years as modulation of the serotonin systems profoundly influence appetite and weight. Enhancing serotonin (i.e., 5-hydroxytryptamine (5-HT)) neurotransmission is known to suppress appetite while dampening serotonin signalling leads to hyperphagia and weight gain [297,298]. The role of the 5-HT_2C_ serotonin receptor in regulating feeding behaviours has been particularly well described [299,300]. Accordingly, 5-HT and 5-HT_2C_ receptor agonists blunt feeding responses in a 5-HT_2C_ receptor antagonist reversible manner [301,302,303,304,305]. Moreover, transgenic mice specifically lacking 5-HT_2C_ receptors are hyperphagic and obese, further substantiating that signalling through this receptor is important for the regulation of appetite [305,306]. 5-HT_2C_ receptor mRNA and protein are ubiquitously found within the brain, including brain region known to regulate feeding [271,272]. Interestingly, its expression within feeding related regions (e.g., HYP), like that of GHSRs, fluctuates with feeding status (i.e., fasted versus fed) with higher 5-HT_2C_ receptor mRNA reported in the fasted state [273]. Moreover, similar to the GHSR, the 5-HT_2C_ receptor, transduces its signal most commonly through a Gα_q_ dependent pathway that ultimately increases intracellular calcium via PLC/IP_3_ signalling cascades [307].

The expression and functional overlap between ghrelin and serotonin systems has provoked research into their potential interaction [13,14,17,271,272]. Most studies have demonstrated that ghrelin and serotonin systems work to antagonize one another but the mechanism by which they do so remains elusive. Accordingly, peripheral administration of 5-HT_2C_ receptor agonists blocks the increase in plasma ghrelin seen following a 24 h fast and increase expression of anorexigenic peptides within the HYP [273]. Conversely, in hypothalamic synaptosomes, activation of GHSRs by ghrelin blocks the release of serotonin [276]. More importantly, intra-PVN microinjections of 5-HT_2C_ receptor agonists (pre-treatment) significantly attenuates the orexigenic effect of intra-PVN ghrelin [67,274,275]. Together these data support a functional antagonistic relationship between ghrelin and serotonin systems.

Although the crosstalk between these systems is not necessarily indicative of a relationship at the receptor level, given the promiscuous nature of the GHSR and overlapping distribution of GHSRs and 5-HT_2C_ receptors, the possibility that they influence one another via heterodimerization cannot be ignored (Table 2) [237,271,272]. Consistent with this, when HEK 293 cells are co-transfected with GHSRs and 5-HT_2C_ receptors these receptors co-localize [236]. Even though both GHSR and 5-HT_2CT_ receptors typically couple to Gα_q_ to elicit increases in intracellular calcium, concomitant treatment with both receptor agonists does not elicit synergistic or additive calcium influx in co-transfected cells relative to administration of either agonist separately [236]. Interestingly, treatment with GHSR agonists significantly increases the co-internalization of these receptors, an effect that is reversed (i.e., increased surface expression) when cells expressing both GHSR and 5-HT_2C_ receptors are treated with a GHSR inverse agonist (i.e., [d-Arg^1^, d-Phe^5^, d-Trp^7,9^, Leu^11^]-substance P) [236]. Accordingly, [d-Arg^1^, d-Phe^5^, d-Trp^7,9^, Leu^11^]-substance P pre-treatment of co-transfected cells enhances serotonin induced calcium influx, an effect noticeably absent in cells co-transfected with just 5-HT_2C_ receptors (indicative of cross sensitization) [236]. The heterodimer pairing between GHSRs and 5-HT_2C_ receptors in co-transfected HEK 293 cells also causes a reduction in GHSR agonist induced GHSR signaling activity [236]. This is exemplified by the 65% reduction in calcium influx following administration of GHSR agonists in cells co-transfected relative to those transfected with the GHSR alone [236]. Furthermore, 5-HT_2C_ receptor antagonist pre-treatment is capable of restoring ghrelin induced calcium influx (equivalent to levels seen in cells only transfected with GHSR) in cells co-expressing GHSR and 5-HT_2C_ receptors [236]. Together, these data highly suggest that GHSR and 5-HT_2C_ receptors are capable of forming heterodimers, a paring that severely hinders the signaling capability normally conveyed by GHSR but does not significantly alter 5-HT_2C_ receptor signaling. It is important that we validate the existence of this heterodimer in ex vivo or in vivo experiments before we begin to speculate on its physiological relevance. Nonetheless, given the prevalence of the 5-HT_2C_ receptor in feeding related brain regions known to house GHSR and the impact that 5-HT_2C_ receptor antagonists and agonists have on feeding behaviours, it remains a promising avenue of exploration.

### 5.6. GHSR/Melanocortin Receptor 3 Heterodimers

The role that MC3-Rs have in the regulation of feeding is slightly more ambiguous as some report hypophagia in MC3-R KO rodents whilst others report no changes in feeding behaviour [308,309,310]. Moreover, MC3-Rs seem to play an important role in entrainment of anticipatory activity precluding meals as MC3-R KOs show deficits in anticipatory behaviours relative to WT animals [310]. Although it was originally proposed that activation of MC3-Rs suppressed feeding primarily due to the use of nonspecific melanocortin agonists that also stimulated anorexigenic MC4-Rs; studies conducted with more specific MC3-R agonists suggest that MC3-R activation promotes appetite [311,312,313,314,315,316]. Accordingly, administration of specific MC3-R antagonists decreases feeding in rodents [314]. Mechanistically, activation of MC3-Rs, located predominantly on POMC neurons within the ARC (but also found on AGRP neurons), acts to dampen POMC neuron activity to promote orexigenic pathways [312,313,315,316,317,318]. Likewise, it was recently demonstrated that activation of VTA MC3-Rs enhances the motivational drive to obtain palatable foods and therefore may likewise contribute to the orexigenic effects observed following MC3-R agonist administration [277].

Investigations into whether GHSR and MC3-R form heterodimers led to the realization that GHSRs and MC3-Rs demonstrate considerable in vivo expression overlap, particularly within the ARC [260]. Combined in situ hybridization and immunohistochemistry experiments reveal that a vast majority of GHSR expressing cells within this region also express MC3-Rs [260]. Generally, activation of MC3-Rs stimulates cAMP production via Gα_s_ dependent signaling [318]. Co-transfection of GHSRs with MC3-Rs in COS-7 cells potentiates α-MSH induced cAMP accumulation about two fold more than those observed when the MC3-R is transfected alone [260]. Conversely, co-transfection of these receptors in HEK 293 cells reduces basal as well as induced canonical GHSR signaling (i.e., over 50% reduction) [236,260]. The potentiation of α-MSH induced cAMP accumulation conveyed by the GHSR/MC3-R is dependent on GHSR constitutive activity as transfection of mutants that do not demonstrate basal activity or treatment with GHSR inverse agonists completely abolish this effect [260]. Interestingly, the heterodimer is found preferentially in the cytosol of HEK-cells co-transfected with both receptors, an effect that is not seen when the receptors are expressed on their own [236]. The internalization of the heterodimer does not rely on GHSR constitutive activity and becomes more prevalent when these cells are treated with GHSR but not MC3-R agonists [236]. These molecular studies present strong evidence that GHSRs and MC3-R form heterodimers; however, confirmation of this heterodimer in vivo and its functional significance are required.

Although behavioural studies probing the relevance of this heterodimer have not been conducted, there are observations that these two systems interact to influence feeding behaviours (Table 2). First, these receptors are both expressed in feeding related brain regions and co-localize on the same cells at least within the HYP [13,14,17,277,278]. Indirect evidence suggest that they may also co-localize in the VTA as MC3-Rs are expressed on VTA dopamine neurons and 50%–60% of VTA dopamine neurons are known to express GHSRs [1,17,277]. Second, independent stimulation of either system is known to enhance both homeostatic and hedonic feeding behaviours [1,10,22,101,113,277,312,314,315]. Lastly, the orexigenic capability of peripheral ghrelin in WT animals is lost in MC3-R KO animals [280]. Taken alone, the above mentioned observations do not overwhelming suggest a functional relevance for the GHSR/MC3-R heterodimer; however, in combination with the GHSR/MC3-R in vitro data, it appears that that the physiological relevance of this heterodimer warrants further exploration, particularly in relation to its role in regulating feeding.

## 6. Conclusions

Since the discovery of the GHSR and its endogenous ligand our understanding of the ghrelin system has evolved immensely. Although ghrelin induced growth hormone release was the first described event following activation of GHSRs, it is now known that the ghrelin system is intimately involved in regulating many physiological processes (e.g., feeding, mood, adiposity, memory). Ghrelin’s influence over these physiological processes and behaviours were largely determined by administering modulators (i.e., agonists or antagonists) of GHSR signaling in GHSR rich brain regions of known function. Interestingly, although exogenous ghrelin clearly has the capacity to activate GHSR within many of these BBB protected brain regions to alter behaviour; the ability of endogenous ghrelin to do so has been strongly questioned. As delineated in this manuscript these reservations stem from the fact: that ghrelin is predominantly activated and secreted from the stomach, that an extremely low percentage of ghrelin circulates in its active acylated form capable of stimulating GHSRs, and that ghrelin has poor BBB penetrance and thus does not seem to be able to access many brain regions known to house GHSRs. Although, the ghrelin system regulates various physiological processes its ability to manipulate feeding behaviours is by far the best characterized. Nonetheless, how the ghrelin system influences feeding processes given the apparent enigmatic spatial separation of GHSR and its endogenous ligand has been largely ignored. The goal of this paper was to address this deficiency and present the most likely means by which the ghrelin system modulates feeding.

Although it is often favoured to think of hormones acting in a homogenous fashion and/or within discrete brain regions, the complexity of behaviours (particularly ones integral to survival such as feeding) do not conform to these simplifications. In reality, it is increasingly apparent that the ghrelin system modulates feeding behaviours in a multifaceted manor. It is clear that peripheral ghrelin can stimulate both central and peripheral GHSRs to transduce orexigenic signals that drive feeding behaviours. Moreover, although hotly contested, evidence of central ghrelin synthesis/activation suggest that feeding behaviour may be locally regulated, at least within the HYPs. Lastly, although in its infancy, the study of ghrelin independent activity of GHSR and its influence over the regulation of feeding behaviours is emerging.

It appears that peripherally produced ghrelin predominantly exerts its orexigenic activity via two main avenues. First, via stimulation of GHSRs located on vagal afferents (which conveniently reside in close proximity to the main site of acylated ghrelin production) and subsequent relay of orexigenic signal from the NTS to the HYP. Second, via activation of the well described GHSR dependent orexigenic circuit that originates in the ARC. Although the access of ghrelin into the ARC has long been debated, recent studies demonstrating nutrient dependent changes in fluorescently labelled ghrelin binding with the ARC convincingly support the ARC as an accessible brain region to peripheral ghrelin. Although CVOs have been suspected entry sites for peripheral ghrelin, it does not seem that peripheral ghrelin targets CVOs to gains access to GHSR rich brain within the CNS (aside from the ME). Nonetheless, it appears that orexigenic signals can be produced in some CVO, most convincingly in the AP, and then transmitted to other feeding related brain regions to promote feeding behaviours.

The notion of central ghrelin synthesis and/or activation has been through rigorous testing given its revolutionary implications. Accordingly the central production of ghrelin would have many benefits over the peripheral production of ghrelin including bypass of the BBB, decreased degradation of acylated ghrelin, and increased spatial and temporal control. Currently, the jury is still out on central ghrelin synthesis as it has not be indubitably demonstrated or discredited. As mentioned, validated antibodies that creditably target acylated ghrelin and better designed ghrelin reporter transgenic lines are desperately needed to resolve debated concerning the central synthesis of ghrelin.

Lastly, and perhaps the most important realization is the idea that GHSR ligand independent activity may regulate the vast majority of processes that up until now have been attributed to ghrelin dependent activation of GHSRs. The notion of GHSR ligand independent activity provides a reasonable explanation as to why GHSRs are so widely distributed in brain regions seemingly inaccessible to ghrelin. Furthermore, the added complexity of ghrelin independent GHSR activity may help rationalize commonplace disparities in both in vitro and behavioural experiments. In addition to the high constitutive activity of GHSRs, they also have the capacity to form heterodimer with at least 5 other GPCRs known to regulate feeding neurocircuits. Research into the molecular and physiological ramifications of these heterodimers in vivo will most likely reveal previously unknown intricacies that influence feeding behaviours.

Ultimately, it is evident that the regulation of feeding behaviours by the ghrelin system is far more complex than originally anticipated. Not only may ghrelin act and be produced both peripherally and centrally, but GHSRs themselves, as well as in concert with other GPCRs possess the ability to convey significant feeding related information. Although it will take years before these complex relationships are disentangled, the knowledge and novel therapeutic strategies uncovered along the way will irrefutably be worth the effort.

## Figures and Tables

**Table 1:** Supporting and refuting evidence regarding the central synthesis of ghrelin.

	Technique(s)	Findings Summary	Species	Reference(s)
**Supporting Evidence**	RT PCR	Ghrelin transcript detected in the brain	Rat	[20]
	RT PCR	Ghrelin transcript detected in the arcuate nucleus (ARC)	Rat	[24]
	RT PCR	Ghrelin transcript detected in the hypothalamus (HYP) of wild-type (WT) but not ghrelin knockouts (KOs)	Mouse	[22]
	RT PCR	Ghrelin mRNA detected in HYP	Rat	[215]
	RT PCR	Ghrelin mRNA detected in cerebral cortex and HYP	Rat	[214]
	RT PCR	Hypothalamic expression of ghrelin significantly lower in *clock* mutants	Mouse	[217]
	RT PCR	Hypothalamic expression of ghrelin and ghrelin *O*-acyltransferase (GOAT) mRNA increases following a 48 h fast	Rat	[56]
	RT PCR and transgenics	Detected ghrelin and enhanced green fluorescent protein (EGFP) transcripts in the HYP of transgenic mice that were engineered to express EGFP in cells that produce ghrelin (i.e., inserted the gene for EGFP in the regulatory region of the ghrelin gene)	Mouse	[225]
	Immunohistochemistry	Ghrelin-immunoreactive neurons detected in the ARC	Rat	[20,24,220,221,223]
	Immunohistochemistry	Numerous ghrelin immunoreactive cell bodies and axons found within the HYP;small number of ghrelin immunoreactive processes found within extrahypothalamic brain regions (e.g., BNST, NA, cortex)	Mouse	[22]
	Immunohistochemistry	Detected ghrelin immunoreactivity in the HYP	Human	[224]
	Immunohistochemistry	Ghrelin immunoreactive neurons were detected in the HYP and cerebral cortex	Rat	[214]
	Immunohistochemistry	HYP ghrelin was detected in sham operated rats but not in those that underwent a pinealectomy	Rat	[220]
	Immunohistochemistry and transgenics	EGFP fluorescence detected in ARC of transgenic ghrelin reporter mice (inserted the gene for EGFP in the regulatory region of the ghrelin gene)	Mouse	[225]
	Reverse phase high performance liquid chromatography and ghrelin radioimmuneassays	Detected ghrelin radioimmunoreactivity in the ARC	Rat	[24]
	Reverse phase high performance liquid chromatogra-phy and ghrelin radioimmuneassays	Detected ghrelin radioimmunoreactivity in the HYP	Rat	[215]
	Behavioural tests	Chronic central GOAT knockdown (via ICV infused amorpholino antisense oligonucleotides) significantly decreased weight gain of rats fed a high fat diet	Rat	[56]
**Refuting Evidence**	RT PCR and transgenics	Failed to detect ghrelin immunoreactivity or ghrelin mRNA (via in situ hybridization) within the brain of WT or ghrelin-hrGFP BAC transgenic reporter mice	Mouse	[25]
	Immunohisto-chemistry	Strong ghrelin immunoreactive signals from the stomach but no specific immunoreactivity for either ghrelin or des-acyl ghrelin in the central nervous system (e.g., HYP, medulla oblongata, and spinal cord) using four separate well characterized anti-ghrelin antibodies	Mouse and Rat	[23]
	Immunohisto-chemistry and transgenics	Evident β-galactosidase staining within the stomach and intestine but no positive staining within the HYP of transgenic ghrelin deficient mice (*ghrl^−/−^*) engineered to express a *lacZ* reporter gene in the place of the functional ghrelin gene WT and ghrelin deficient mice show negligible and indistinguishable hypothalamic ghrelin immunoreactivity despite having very different stomach ghrelin immunoreactivity profiles	Mouse	[218]
	Immunohisto-chemistry and transgenics	Failed to detect GFP fluorescence in the brain but demonstrated clear GFP expression in the stomach using two separate transgenic ghrelin reporter mice (i.e., ghrelin-hrGFP BAC transgenic mice), in which humanized *Renilla reniformis* GFP expression was driven by different lengths of the ghrelin promotor	Mouse	[25]

**Table 2:** Summary of GHSR-1a heterodimers and their putative implication in regulating feeding behaviours.

Heterodimer	In Vitro Observations of Heterodimer	Ex Vivo Observations of Heterodimer	Feeding Related Brain Regions Where Receptor Expression Overlaps	Known Cross Talk Between Systems	Influence of Heterodimer Over Feeding Behaviours
**GHSR-1a/GHSR-1b**	Low ratio of GHSR-1b relative to GHSR-1a ↑cell surface expression of the heterodimer and↑Gα_i/o_ dependent signaling cascades following ghrelin administration [258] High ratio of GHSR-1b relative to GHSR-1a↓cell surface expression of the heterodimer and↓Gα_i/o_ dependent signaling cascades following ghrelin administration [258]	Increasing the expression of GHSR-1b in striatal (i.e., naturally high GHSR-1b to GHSR-1a expression ratio) cultures↓ghrelin signalling efficiency [258] Increasing the expression of GHSR-1b in hippocampal (very low inherent GHSR-1b/GHSR-1a expression ratio) cultures↑ghrelin signalling efficiency [258]	HIP, NA [12,13,14,259]	If GHSR-1b subunit is much higher than GHSR-1a then the GHSR-1a/GHSR-1b heterodimer can still bind agonists but forfeits the capacity to stimulate associated signaling proteins [257]	Unknown but speculated↑in feeding when ratio of GHSR-1a to GHSR-1b is high but↓in feeding when ratio of GHSR-1a to GHSR-1b is low
**GHSR-1a/DRD1**	Co-localize, immuno-precipitate together, and give off a reliable positive bioluminescence resonance energy transfer signal only in the presence of agonists (i.e., dopamine and ghrelin) [234] Enhances Ca^2+^ and cAMP accumulation in HEK cells in response to ghrelin and dopamine above those observed when GHSR or DR1D are expressed alone [234]	DRD1 agonists significantly enhance the formation of GHSR/DRD1 heterodimers within hippocampal neurons [157] DRD1 and GHSR agonists enhance Ca^2+^ in WT neurons that express the heterodimer but are unable to elicit this effect in GHSR antagonist treated and GHSR KO hippocampal neurons [157]	HIP, VTA, Striatum, Cortex [12,13,14,235]	Pre-treatment with a D1-like antagonist into the NA, completely blocks the rewarding effects of intra-VTA ghrelin [116] GHSR inactivation completely attenuates DRD1 regulated hippocampal behaviour and memory [256]	Unknown
**GHSR-1a/DRD2**	DRD2 agonists↑intracellular calcium levels in HEK 293 cells which co-express both GHSR-1as and DRD2s but not in cells transfected with either receptor independently [235] HEK 293 cells that are pre-treated with either DRD2 or GHSR antagonists do not enhance intracellular calcium levels in response to DRD2 or GHSR agonists [235]	FRET detection of GHSR-1a/DRD2 heterodimers in hypothalamic preparations of WT but not GHSR KO mice [235]	HIP, HYP, Striatum [12,13,14,236]	DRD2 agonists known to suppress appetite, reliably↓food intake in WT but not GHSR KO mice [235] Pre-treatment of WT mice with GHSR antagonists, prevent decreases in feeding following cabergoline injections [235] Pre-treatment with a D2-like antagonist into the NA, completely blocks the rewarding effects of intra-VTA ghrelin [116]	Unknown but speculated attenuation of feeding behaviours
**GHSR-1a/5-HT_2C_**	When GHSR-1as and 5-HT_2C_ receptors are co-transfected into HEK 293 cells they co-localize [236] GHSR-1a agonists significantly↑the co-internalization of these receptors in HEK 293 cells [236] Co-transfection of GHSR-1a and 5-HT_2C_ in HEK 293 cells reduces GHSR agonist induced GHSR signaling activity (65% in Ca^2+^ mobilization) [236]	Unknown	HIP, HYP, VTA, Cortex [12,13,14,271,272,273]	Peripheral administration of 5-HT_2C_ receptor agonists block the↑in plasma ghrelin seen following a 24 h fast and increases expression of anorexigenic peptides within the HYP [273] Intra-PVN microinjections of 5-HT_2C_ agonists significantly attenuates the orexigenic effect of intra-PVN ghrelin [274,275] GHSR activation by ghrelin blocks the release of serotonin in hypothalamic synaptosomes [276]	Unknown but speculated attenuation of feeding behaviours
**GHSR-1a/MC3-R**	Co-transfection of GHSRs and MC3-Rs in COS-7 cells potentiates α-MSH induced cAMP accumulation [260] Co-transfection of these receptors in HEK 293 cells↓basal as well as ghrelin induced canonical GHSR signaling (i.e., >50%↓) [260]	Unknown	HYP, VTA [12,13,14,265,266,277,278]	The orexigenic effects of peripheral ghrelin in WT mice is lost in MC3-R KO mice [279]	Unknown but speculated↑in feeding
